# Super-Resolution Microscopy and Single-Molecule Tracking Reveal Distinct Adaptive Dynamics of MreB and of Cell Wall-Synthesis Enzymes

**DOI:** 10.3389/fmicb.2020.01946

**Published:** 2020-08-20

**Authors:** Simon Dersch, Johanna Mehl, Lisa Stuckenschneider, Benjamin Mayer, Julian Roth, Alexander Rohrbach, Peter L. Graumann

**Affiliations:** ^1^SYNMIKRO, LOEWE-Zentrum für Synthetische Mikrobiologie, Philipps-Univetsität Marburg, Marburg, Germany; ^2^Fachbereich Chemie, Philipps-Univetsität Marburg, Marburg, Germany; ^3^Laboratory for Bio- and Nano-Photonics, Department of Microsystems Engineering-IMTEK, BIOSS Centre for Biological Signalling Studies, University of Freiburg, Freiburg, Germany

**Keywords:** cell shape maintenance, MreB cytoskeleton, *Bacillus subtilis*, RodA, single molecule dynamics

## Abstract

The movement of filamentous, actin-like MreB and of enzymes synthesizing the bacterial cell wall has been proposed to be highly coordinated. We have investigated the motion of MreB and of RodA and PbpH cell wall synthesis enzymes at 500 ms and at 20 ms time scales, allowing us to compare the motion of entire MreB filaments as well as of single molecules with that of the two synthesis proteins. While all three proteins formed assemblies that move with very similar trajectory orientation and with similar velocities, their trajectory lengths differed considerably, with PbpH showing shortest and MreB longest trajectories. These experiments suggest different on/off rates for RodA and PbpH at the putative peptidoglycan-extending machinery (PGEM), and during interaction with MreB filaments. Single molecule tracking revealed distinct slow-moving and freely diffusing populations of PbpH and RodA, indicating that they change between free diffusion and slow motion, indicating a dynamic interaction with the PGEM complex. Dynamics of MreB molecules and the orientation and speed of filaments changed markedly after induction of salt stress, while there was little change for RodA and PbpH single molecule dynamics. During the stress adaptation phase, cells continued to grow and extended the cell wall, while MreB formed fewer and more static filaments. Our results show that cell wall synthesis during stress adaptation occurs in a mode involving adaptation of MreB dynamics, and indicate that *Bacillus subtilis* cell wall extension involves an interplay of enzymes with distinct binding kinetics to sites of active synthesis.

## Importance

The shape of bacterial cells is dictated by the shape of the cell wall, a peptidoglycan (PG) polymer. The polymer is synthesized by the combined activity of transglycosylases (TG) that extend PG strands and transpeptidases (TP) that crosslink strands. Using a combination of superresolution-microscopy and single molecule tracking, we show that in *Bacillus subtilis*, a TG and a TP enzyme show similar directional movement when involved in PG synthesis, but different lengths of times for this slow movement. Filament-forming, membrane-associated protein MreB, which is also essential for proper cell wall architecture, moves in parallel with TPs and TGs underneath the cell membrane, but with longer persistence than TGs and TPs, indicating that the enzymes transiently interact with MreB filaments. At a single molecule level, MreB, RodA, and PbpH show different dynamics, especially during a phase of adaptation after environmental stress, where TG and TP continue their regular dynamics, while MreB becomes more freely diffusive and forms fewer filaments that are less mobile. While cell growth continues, the patterns of incorporation of new cell wall material becomes more irregular, indicating that MreB filaments are not required for cell wall extension *per se*, but likely contribute to the accuracy of the pattern of synthesis.

## Introduction

Bacteria have evolved an enormous assortment of different shapes to adapt to vastly different niches (Yang et al., 2016). In recent years, it has become clear that a multitude of proteins affect cell morphology: not only do enzymes that actively extend the existing peptidoglycan (PG) strands hugely impact cell shape (Cabeen and Jacobs-Wagner, 2005), but also proteins that affect ionic conditions within the wall (i.e., proteins generating teichoic acids within the cell wall) (Schirner et al., 2009), proteins that form filamentous structures within the cell (actin-like MreB) (Graumann, 2007), and also proteins that provide precursors of cell wall material. It appears that an intricate interplay exists between various intra- and extracellular synthetic enzymes, which may be coordinated by an apparent scaffold of MreB filaments underneath the cell membrane.

MreB has been intensively investigated. Initially it was presumed that MreB (as well as its two paralogous proteins in *B. subtilis* Mbl and MreBH) form long, helical filamentous structures, that localize circumferentially along the cell periphery (Errington, 2015). Utilizing advanced fluorescence microscopy techniques like total internal reflection fluorescence microscopy (TIRFM), super-resolution structured illumination (SIM) and stimulated emission depletion (STED), MreB has been identified to localize in much smaller assemblies with varying lengths, from a few hundred nanometers up to micrometers (although there has been recent evidence against very long structures, Billaudeau et al., 2019), that processively move along tracks mostly perpendicular to the long axis of the cell (Dominguez-Escobar et al., 2011; Olshausen et al., 2013). It has been suggested that the intrinsic curvature of MreB filaments is responsible for its circumferential localization pattern and its observed preference for regions with the greatest principal membrane curvature, which could guide the correct orientation of the enzymes of the cell wall elongasome responsible for cell wall synthesis (Hussain et al., 2018). According to a popular view, MreB provides a coordination of cytosolic enzymes that synthesize the cell wall precursor β-(1,4) linked *N*-acetylglucosamine/*N*-acetylmuramic acid (a disaccharide connected to a five amino acids polypeptide) with the motion of membrane proteins that synthesize the cell wall on the other side of the cell membrane. Such enzymes include RodA, a major peptidoglycan polymerase (Meeske et al., 2016; Emami et al., 2017), bifunctional Penicillin-binding proteins (PBPs) that contain glycosyl transferase activity (i.a. PG polymerization) and amino acid transferase activity, monofunctional PBPs that solely perform the amino acid transferase step that crosslinks the glycan strands, and cell wall hydrolases. Indeed, MreB physically interacts with PBPs, RodA, MreC, and MreD membrane proteins (Figge et al., 2004; Defeu Soufo and Graumann, 2006; Kawai et al., 2009), as well as with MurG and other precursor-synthesis enzymes (Mohammadi et al., 2007; White et al., 2010; Favini-Stabile et al., 2013). Moreover, the motion of some cell wall synthetic enzymes has been shown to parallel that of MreB filaments using live cell fluorescence microscopy (Dominguez-Escobar et al., 2011; Garner et al., 2011; Morgenstein et al., 2015; Cho et al., 2016). Interestingly, when cell wall synthesis is inhibited, the motion of MreB filaments becomes highly reduced (Dominguez-Escobar et al., 2011; Garner et al., 2011; van Teeffelen et al., 2011), which has led to the model that perpendicular movement of MreB filaments is driven by the polymerization activity of cell wall synthetases.

More recent studies of MreB have used particle tracking methods and computational analysis to identify patterns of motion. In both *E. coli* and *B. subtilis* various subpopulations of MreB could be differentiated, some following helical tracks, while others appear to move more randomly (Lee et al., 2014; Billaudeau et al., 2017). It has also been observed that *B. subtilis* might regulate the speed of MreB structures in response to an upshift in nutrient availability, while in *E. coli* speeds did not vary significantly, but the distribution of subpopulations shifted, suggesting differing mechanisms of adaption in gram positive and negative organisms (Billaudeau et al., 2017).

Curiously, the presence of MreB in cells is essential, but can be avoided when magnesium levels in the medium are increased to higher than usually used levels for growth in *B. subtilis* (Formstone and Errington, 2005). It has also been shown that the three *mreB* paralogous genes in *B. subtilis*, *mreB*, *mbl*, and *mreBH*, have overlapping functions, and that a triple deletion can be achieved by an upregulation of the sigma I transcription factor that responds to cell envelope stress (Schirner and Errington, 2009). This connection between MreB and regulation of cell wall stress response has not been investigated in much detail yet.

We wished to analyze and compare the movement of MreB filaments and that of two important peptidoglycan synthesis enzymes, RodA and PbpH (class B monofunctional transpeptidase), at high spatiotemporal resolution, by using a combination of Total Internal Reflection Fluorescence – Structured Illumination Microscopy (TIRF-SIM) and single-molecule tracking (SMT). This allowed us to follow the movement of molecules at two different times scales: TIRF-SIM visualizes the ensemble movement of polymerized MreB molecules and of several RodA and PbpH molecules at 2 frames per second, revealing the dynamics of entire MreB filaments, while SMT follows the movement of single molecules at 50 frames per second, which reveals even freely diffusive motion. We also reasoned that conditions of optimal growth may not reflect the entire repertoire of MreB dynamics. Because of the genetic link between *mreB* deletion and magnesium homeostasis in the cell, we analyzed protein and cell wall synthesis patterns and dynamics in cells that were osmotically stressed, uncovering that especially under these conditions, the motion of cell wall synthesis enzymes and that of MreB strongly deviates.

## Results

### TIRF-SIM Tracking Shows That MreB, RodA and PbpH Move Along Similar Trajectories With Similar Velocity

MreB assembled into filaments has been shown to processively move underneath the cell membrane, along trajectories that run in both, opposing orientations, mostly perpendicular to the short axis of rod shaped cells. MreB movement has been correlated to an apparently similar movement of enzymes involved in cell wall synthesis (Dominguez-Escobar et al., 2011; Garner et al., 2011; van Teeffelen et al., 2011). We wished to gain a clearer view on the dynamics of the molecules using superresolution (SR) fluorescence microscopy, with the specific question whether MreB filament dynamics indeed follow similar paths and have similar dynamics compared to RodA and PbpH cell wall synthesis enzymes, and if MreB, RodA, and PbpH behave similarly at the single molecule level.

**FIGURE 1:**
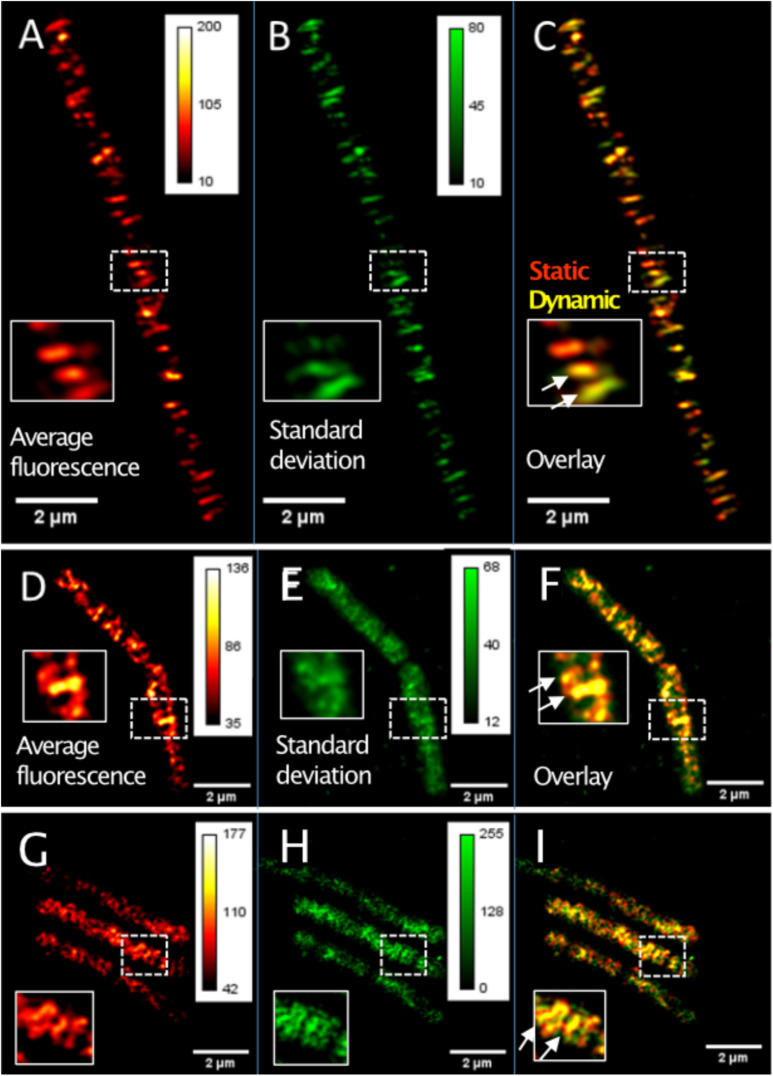
TIRF-SIM analysis of exponentially growing cells expressing functional GFP-MreB YFP-RodA and YFP-PbpH fusions from ectopic sites on the chromosome. **(A)** Time-averaged intensity of 20 subsequent images (time projection) of MreB filament dynamics, **(B)** corresponding standard deviation (time projection) of MreB filaments, **(C)** overlay of time projection and standard deviation, revealing static (red) and dynamic (yellow) filaments. Note that there is a space window of *L*_*max*_ = 800 nm, due to the TIRF-illumination and a corresponding time window of *t_*max*_ = L/v*. **(D)** Average intensity of 20 subsequent images (time projection) of YFP-RodA dynamics, **(E)** corresponding standard deviation (time projection) for YFP-RodA, **(F)** overlay (see **C**), **(G)** average intensity of 20 subsequent images (time projection) of YFP-PbpH assemblies, **(H)** corresponding standard deviation (time projection), **(I)** overlay. Heat maps show fluorescence intensities as arbitrary units.

TIRF-SIM can reveal protein dynamics at a spatial resolution of about 120 nm and in the range of few seconds for temporal resolution, while SMT has a temporal resolution in the milliseconds range. We imaged GFP-MreB [or YFP-MreB for SMT, these fusions have been shown to functionally replace wild type MreB in Reimold et al. (2013)] in 0.5 s intervals, using a custom built setup (Roth et al., 2020). In order to avoid overproduction artifacts, the fusion was expressed at very low levels (0.01% xylose) from an ectopic site on the chromosome, such that cells express very few GFP-MreB molecules besides wild type MreB protein (Supplementary Figure S1A). Such cells showed rod cell shape indistinguishable from that of cells that did not carry an additional copy of *gfp-mreB* (Supplementary Figure S1C) (Dominguez-Escobar et al., 2011; Reimold et al., 2013). Supplementary Figure S2A shows a typical single TIRF-SIM image of GFP-MreB filaments. Figure 1A shows an average (time projection) over 20 subsequent TIRF-SIM images, indicating all positions of MreB proteins during a time window of 20 s, whereas the time standard deviation in Figure 1B indicates the average changes in these positions and thereby the dynamics of MreB filaments. The red-green overlay shown in Figure 1C illustrates the changes over time, revealing MreB filaments that move or are statically positioned.

From 100 trajectories analyzed through their kymographs, we found a velocity of v = 37 ± 16 nm/s for the movement of MreB filaments (note that ectopically expressed GFP-MreB dynamics closely resemble those of GFP-MreB expressed from the original gene locus, Olshausen et al., 2013). The trajectory angle was around θ = 88 ± 8°, trajectory length observed was 469 ± 261 nm (an example of time resolved movement is shown in Supplementary Figure S2D). These data are in agreement with earlier reports on the dynamics of MreB filaments (Garner et al., 2011; Billaudeau et al., 2019). It has to be considered that the observation of tracks longer than *L*_*max*_ = 800 nm was not possible due to the space-time window of the evanescent illumination of the sample.

As reported before (Olshausen et al., 2013), we observed non-continuous movement of MreB filaments, such as stop and go behavior or reversal (Supplementary Movie S1). Filaments with stop and go behavior move along their trajectories and stop their movement after some time for yet unknown reasons. Filaments with reversal behavior move along a trajectory and change their direction after some time. In 100 trajectories analyzed 20% were stop and go filaments, while only 5% showed reversal behavior.

A major goal of this study was to monitor the movement of MreB and of enzymes that synthesize the cell wall with the same SR microscopy setup (Rohrbach lab). RodA has recently been identified as a major driver of peptidoglycan synthesis (Meeske et al., 2016; Emami et al., 2017). We generated a GFP-RodA and a YFP-RodA fusion that was integrated at the original gene locus by single crossover. The first 500 bp of the *rodA* gene were used to create the N-terminal fusion, such that the original promoter drives expression of a truncated *rodA* gene, while the GFP- or YFP-RodA fusion is driven by the xylose promoter. We used 0.1% xylose as level of induction, because this resulted in the generation of rod shaped cells with wild type morphology (Supplementary Figure S1E), while lower levels led to the formation of round cells (data not shown). A similar fusion has been described to be able to functionally replace the wild type protein (Dominguez-Escobar et al., 2011). Although we cannot rule out that the protein fusion is somewhat overproduced, it retains functionality to replace the wild type protein.

Different from the filamentous structures seen for YFP-MreB (Supplementary Figure S2A), YFP-RodA localized as discreet, diffraction-limited signals with no more than 120 nm in diameter (this is the resolution in x/y of >TIRF-SIM) (Supplementary Figure S2E). Please note that single SIM images are shown in Supplementary Figures S2A,E,H, while Figures 1A,D,G show time averaged fluorescence of several SIM images. From the latter, it can be seen that many YFP-RodA spots moved perpendicular to the long axis of the cells (Figure 1D). A majority of RodA signals moved along a trajectory at the cell periphery for a few seconds and then disappeared, as demonstrated by the homogeneous intensity projection in Figure 1D. Only a minority appeared for a few seconds without moving and then disappeared, resulting in a speckle-like distribution of the standard deviation (Figure 1E). Of note, movement occurred in both directions relative to the long axis of the cells, within the same cell, showing that similar to MreB filaments, RodA assemblies can move with opposing directionality. The velocities of the RodA signals were *v* = 33 ± 16 nm/s and the trajectory angles were θ = 88 ± 10°. Similar to MreB, we observed stop- and go events or reversals of movement (Supplementary Figure S2L), however, fewer than seen for MreB. Our data are comparable to findings on directed movement of RodA and Pbp2 in *E. coli* cells (Cho et al., 2016), and of RodA in *B. subtilis* (Dominguez-Escobar et al., 2011).

As a second representative of cell wall enzymes, we tracked YFP-PbpH, which also has been reported to constitute a functional fusion protein (Dominguez-Escobar et al., 2011), also expressed at low level from the original gene locus. The fusion was constructed analogous to that of YFP-RodA; thereby, YFP-PbpH expression is driven by the xylose promoter, as sole source of the protein in the cell. Cells expressing this fusion and carrying a deletion of the gene encoding for Pbp2a (*pbpA*) had wild type morphology (data not shown). Because a deletion of both, *pbpH* and *pbpA* genes is lethal (Wei et al., 2003), this suggests that the YFP-PbpH fusion can functionally replace the wild type fusion. Supplementary Figure S2H shows that similar to RodA, YFP-PbpH appeared distributed in discreet round or elongated structures along the surface of the cell, which moved along trajectories that were mostly perpendicular (Figures 1G–I), similar to MreB and RodA. Similar to RodA, YFP-PbpH spots either moved and then disappeared, were static and disappeared, and showed noticeable reversals or stop and go motion (Supplementary Figure S2K). The velocities of the PbpH structures were *v* = 38 ± 22 nm/s and the trajectory angles were θ = 92 ± 14°.

When compared with each other (Supplementary Figure S3), all three proteins showed preferential movement along 90°, with PbpH having the broadest distribution, and moved at similar velocities. Please note that RodA and PbpH do not show normal distributions of velocities. Thus, the mean values of for the velocity distributions only serve as an indicator for the distinct dynamics of MreB, RodA and PbpH that are apparent in the distinct shape of the distributions, as well.

### MreB, RodA and PbpH Show Considerably Different Trajectory Lengths

**FIGURE 2:**
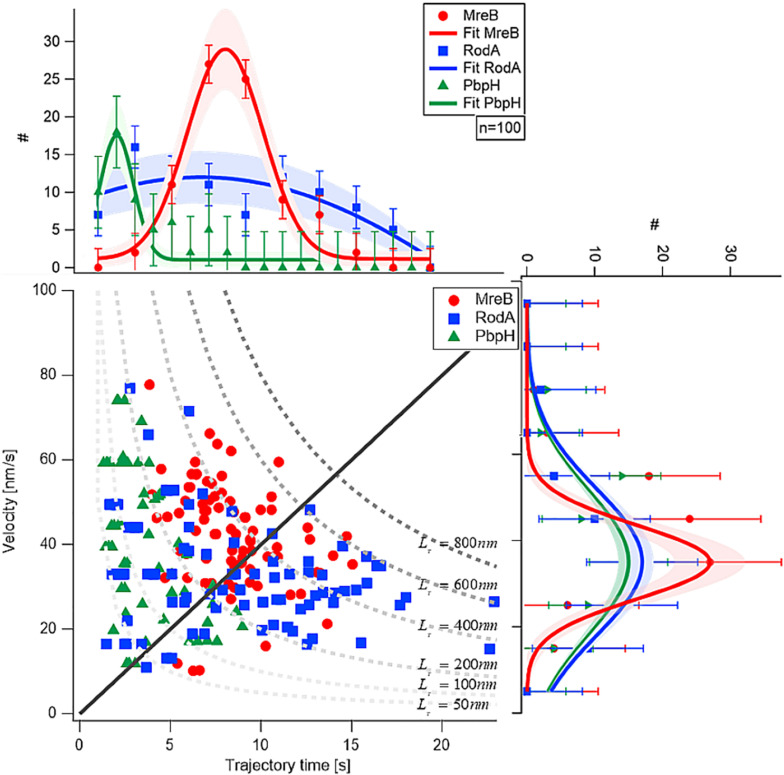
Comparison of velocity and trajectory time distributions of MreB, RodA, and PbpH On the right hand side the similar velocities of MreB, RodA, and PbpH can be seen. The histograms of the protein velocities are shown with their respective Gaussian fit. On the upper part of the figure the histograms of the trajectory times and their respective Gaussian fits are shown. While PbpH has very short trajectory times, RodA and MreB have longer trajectory times. Trajectory lengths (TL) longer than 800 nm were not observed (gray dotted line). Standard deviations of the binned measurements are shown with error bars, the shaded area around the graphs is the error of the Gaussian fit, which is calculated with the square root of the value.

Next, we analyzed the length of the trajectories, i.e., the time for which assemblies or filaments could be observed to move along the perpendicular (or deviating) trajectories. While PbpH moved on trajectories for a relatively short time of approximately 2.4 s and then disappeared, RodA and MreB showed considerably longer trajectory times, approximately 3.8 and 8 s, respectively (Figure 2).

Although MreB was labeled with GFP and PbpH with YFP, the different bleaching speeds cannot be a plausible explanation for the strikingly different trajectory lengths. One has to consider that the proteins were imaged with TIRF-SIM using a 50 mW laser with a wavelength of 488 nm, generating about 1 mW/50 μm^2^ in the sample plane. This gentle excitation of the fluorophores avoided unnecessary bleaching. Considering this, a complete bleach out of the photostable fluorophore YFP after 2–5 s is rather unlikely; faster bleaching of YFP compared to the less stable GFP is also highly unlikely. Thus, we conclude that the observed dynamics are a result of the protein dynamics of PbpH. All measured results are shown in Table 1. From the trajectory lengths and times, we considered the length of disaccharide units inserted with one cell wall precursor having approximately 1 nm, and calculated binding rates $k_{off}\left[Hz\right]\left(k_{off}=\frac{1}{trajectorytime}\right)$. RodA showed a rate of 0.26 ± 0.15 [Hz], and for PbpH, a correspondingly higher off rate of 0.41 ± 0.19 [Hz]. Thus, if indeed RodA and PbpH are in a synthetically active mode while they move directionally, PbpH exchanges more rapidly than RodA.

**TABLE 1:** Dynamic parameters of MreB, RodA and PbpH.

	MreB	RodA	PbpH
Mean ± Std velocity [nm/s]	37 ± 16	33.5 ± 16	38 ± 22
Mean l’ Std trajectory angle [°]	88 ± 8	88.7 ± 9.8	91.8 ± 14
Mean l’ Std trajectory length [nm]	469.5 ± 261	292 ± 202	151.8 ± 66.2
Mean l’ Std trajectory time [s]	8 ± 3	3.8 ± 2.1	2.4 ± 1.1
Mean l’ Std PG-insertions [no.]	470 ± 260	292 ± 202	152 ± 66
Mean l’ Std *k*_*off*_[*Hz*]	–	0.26 ± 0.15	0.41 ± 0.19

Based on the observed similar velocities and angle distributions (Supplementary Figure S3), our experiments support the model that MreB, RodA, and PbpH move together, but do not progress alongside for more than a few seconds, and that RodA and PbpH have different times of interactions with MreB, and with their substrates. This is in agreement with several interaction studies (Kawai et al., 2009; White et al., 2010) showing direct or indirect physical connections between MreB, RodA, and PBPs. The similar velocities at yet differing trajectory times (Figure 2) indicate that RodA and PbpH may hop on and off MreB filaments during exponential growth, and spend different time spans in their cell wall synthesis- mode, i.e., in a synthetically active complex with MreB, and otherwise diffuse within the membrane, which is shown below. Transient interaction times with different RodA and PBP molecules, which may extend peptidoglycan strands in different directions relative to the circumference of the cell, would explain changes of trajectory angles of MreB filaments, and their stop and go periods.

### MreB Single Molecule Dynamics Can Be Explained by 2 Populations With Distinct Diffusion Coefficients

**FIGURE 3:**
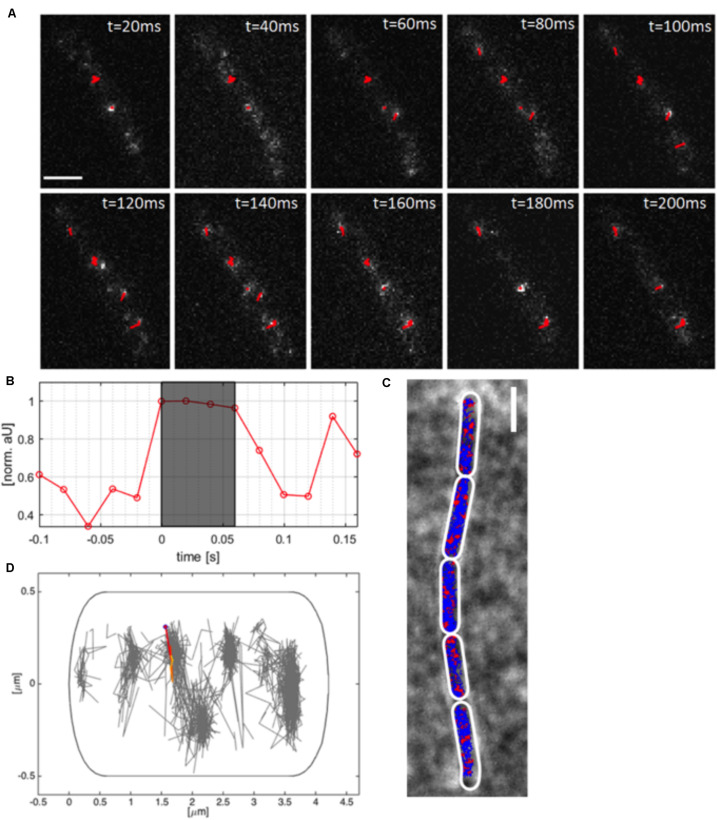
Single molecule tracking of YFP-MreB. **(A)** Tracks (red, containing several steps) of linked sub-pixel localization events, identified by Gaussian-fitting, overlaid on a time-lapse of YFP-MreB (20 ms acquisition time), scale bar 2 μm; **(B)** Relative intensity of identified track as normalized arbitrary units over time; **(C)** Example of a chain of *B. subtilis* cells in exponential phase (*pxyl-yfp-mreB::amyE*,+ 0.01% Xyl) with applied polygonal meshes (white) overlaid with single-molecule tracks of expressed YFP-MreB (blue) and confined tracks (red, 200 nm radius), scale bar 2 μm; **(D)** Distribution of tracks in a single exemplary cell (normalized), single track is highlighted.

Using TIRF-SIM, we gained insight into the dynamics of polymerized MreB molecules, and of RodA and PbpH molecules moving at a similar speed, which are assumed to be actively involved in cell wall synthesis. We wished to gain insight into the percentage of MreB molecules bound within filaments, and of unbound molecules that we assumed to be freely diffusing along the membrane or through the cell. We employed single molecule tracking using 20 ms stream acquisition, such that the movement of diffusive molecules can be visualized, reaching a 25-fold higher temporal resolution than in TIRF-SIM. Our setup of “YFP-bleaching” based SMT avoids blue-light induced inhibition of cell growth (El Najjar et al., 2020), and uses lower laser powers than live PALM. A “slim-field” microscope setup illuminates an area with a diameter of about 15 μm, where initially, fluorescent molecules are bleached until single molecules per cell are visible, which are identified by characteristic single bleaching steps (Figures 3A,B) (Rosch et al., 2018a). U-track (Jaqaman et al., 2008) identifies the centroids of point spread functions via Gaussian-fitting, and links the sub-pixel events, which were classified as tracks if they showed at least four uninterrupted steps (Figure 3B).

**FIGURE 4:**
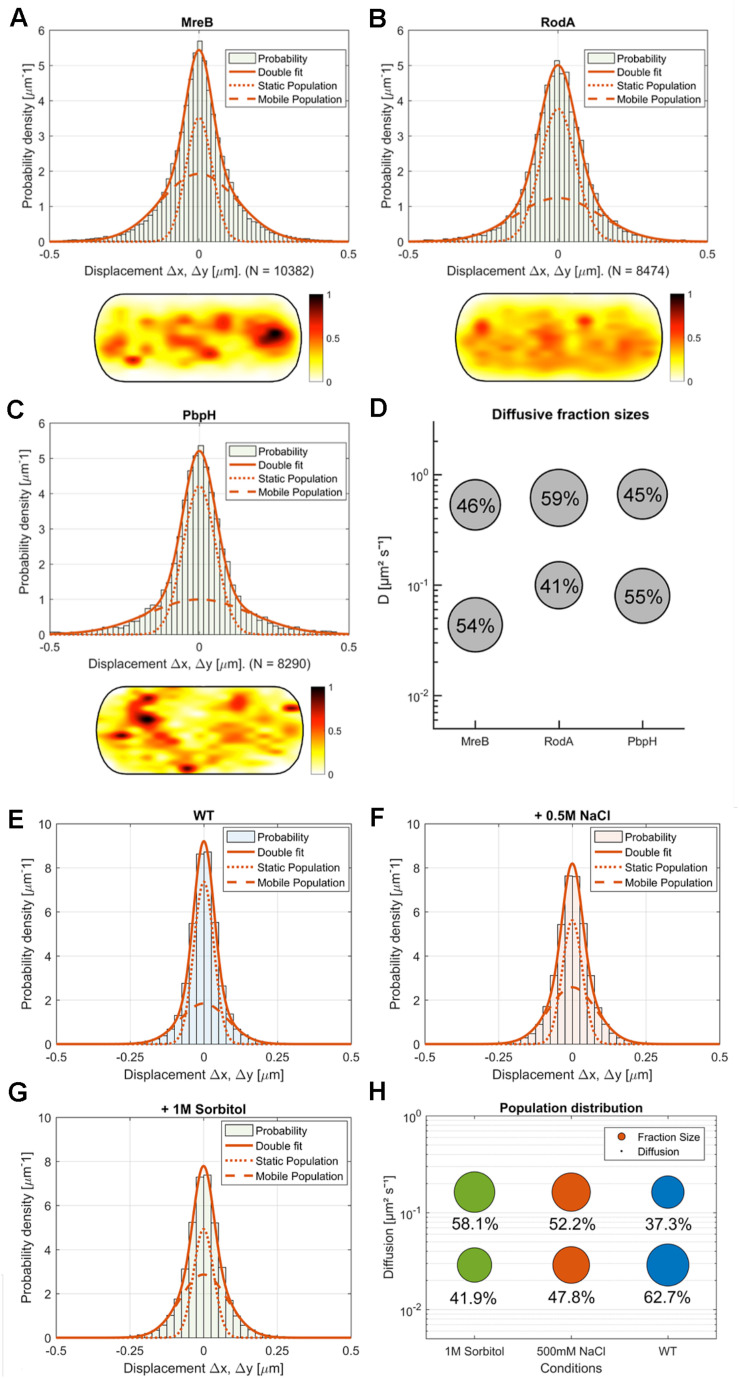
Comparison of MreB, RodA, and PbpH single-molecule motion. **(A–C)** Two-population Gaussian-mixture-model (GMM) fit of **(A)** MreB, **(B)** RodA, **(C)** PbpH displacement vs. probability density and corresponding heat map of the probabilistic distribution of tracks in a normalized cell (dark red: higher probability, white: lower probability); **(D)** Bubble-plot of the diffusive populations (relative fraction sizes, D [μm^2^s^– 1^]) as identified by non-simultaneous GMM curve fit for MreB, RodA and PbpH; **(E–H)** Single-molecule dynamics of GFP-Mbl. expressed under control of the native promotor. Simultaneous two-population Gaussian-mixture-model (GMM) fit of displacement vs. probability density, **(E)** under normal growth, **(F)** with the addition of 0.5 M NaCl, **(G)** with the addition of 1M sorbitol; **(H)** Bubble-plot of the diffusive populations (relative fraction sizes, D [μm^2^s^– 1^]) as identified by GMM curve fit.

As explained above, YFP-MreB was expressed in a merodiploid background, such that very few YFP-MreB molecules were present in a wild type MreB background, allowing that single molecule level was reached very quickly to avoid extended bleaching of cells. An example of MreB tracks of a *B. subtilis* strain expressing YFP-MreB under the inducible xylose promotor (low induction: 0.01% xylose) from an ectopic site on the chromosome can be seen in Figure 3A, and in Supplementary Movie S2. Bleaching of a single molecule that is fluorescent in the gray shaded area to background levels is shown in Figure 3B. Polygonal cell meshes (Figure 3C) were included in Oufti (Paintdakhi et al., 2016), tracks were identified using u-track (Jaqaman et al., 2008), and are shown in blue (non-confined) and red (confined tracks, 120 nm radius) in Figure 3C. We did not allow for any gaps within tracks, average track length was 7 steps, 10% of all tracks were longer than 10 steps, giving us confidence that we can adequately monitor MreB single molecule movement with sufficient statistical reliability. We employed a Matlab-based program called SMTracker (Rosch et al., 2018b) to analyze the obtained tracks. Tracks could be analyzed individually, on a cell to cell basis, or projected onto a standardized cell of 4 × 1 μm (Figure 3D). An example of the generally perpendicular movement, is shown in Figure 3D. For 100 individual cells, around 1000 tracks could be identified, with a localization precision of ∼50 nm (Supplementary Figure S1E). Tracks longer than three times the average lifetime of a fluorophore (3.6 s) were considered outliers and discarded from analyses. Instead of using Mean Squared Displacement (MSD) analysis, which calculates diffusion constants of all observed molecules, we determined diffusion rates from Gaussian mixture model (GMM) curve fits (Figure 4A). GMM analyses can reveal if the probability density function of observed displacements of molecules can be explained by a single population (e.g., one freely diffusive population), or by several populations (e.g., a DNA-bound or diffusive population for a DNA-binding protein). The goodness of the Gaussian fits was determined by comparing observed with predicted data spreads, for all tested condition the *R*^2^ was at least 0.98, indicating sufficient data quality (Supplementary Figure S4E).

We found that MreB diffusion could be best described by at least two existing populations: A fast population (0.53 μm^2^s^–1^ ± 0.08) and a slow-moving population (0.044 μm^2^s^–1^ ± 0.005) (Figures 4A,D). Moreover, our SMTracker tool enables us to compare different methods of obtaining diffusive populations. When we calculated the diffusion via GMM, jump distance analysis or apparent diffusion, we found that in all cases one population would not be sufficient to describe the data (not shown). A two population GMM fit could well explain the data, suggesting that the slow-moving population corresponds to filament-bound MreB, while the faster population likely consists of molecules that freely move along the membrane or through the cytosol. Because MreB is not an integral membrane protein but has a membrane anchor (Salje et al., 2011), it is most likely that the mobile fraction reflects membrane-associated and unbound MreB. The diffusivity of *D* = 0.044 μm^2^s^–1^ ± 0.005 corresponds to a slow-moving membrane protein (Lucena et al., 2018), and is comparable to MreB filaments moving at a speed of 35 nm/s, while 0.53 μm^2^s^–1^ ± 0.08 is in the range of a freely diffusing cytosolic protein (e.g., DnaA, Schenk et al., 2017). The relatively low diffusion constant of MreB could be based on some molecules moving along the membrane, as MreB has membrane-affinity (Salje et al., 2011), or could be based on MreB possibly moving as a multimer (e.g., trimer), which we can not presently distinguish. In comparison to the non-Gaussian distribution of MreB data in Figure 4A, a freely diffusive protein such as Phosphofructokinase (PfkA) shows an almost completely Gaussian distribution (El Najjar et al., 2018). Fitting of the GMM data suggests that 46 ± 10% of the expressed MreB molecules are in the freely diffusive mode, while 54 ± 10% are bound to filaments (Figures 4A,D). When all tracks obtained from many cells are projected into a standardized cells of 3 × 1 μm (average length of cells), regions of how and high density of molecules can be visualized. Such a “heat map” of YFP-MreB molecules (Figure 4A) reveals a concentration toward the center of the cell, due to the fact that molecules were tracked by moving the focal plane toward the top of the cells to best capture the membrane-bound proteins. This may lead to an overestimation of membrane-associated molecules versus cytosolic, freely diffusing molecules. It should also be noted that the diffusion constant for filamentous, slow-moving/membrane-associated MreB must be corrected by a factor of 1.23 in order to account for the curvature of the membrane, as was experimentally determined (Lucena et al., 2018). The true diffusion constant of freely diffusive MreB in the cell is also higher, because movement in the Z-direction is ignored in SMT experiments, because only 2D movement can be captured, unless a special 3D setup is used.

We also tracked a GFP-Mbl fusion that is expressed as sole source of the protein in the cell, from the original gene locus under its native promoter. This fusion is thus expressed at physiological level. Single molecule dynamics of GFP-Mbl were in the same range as those of YFP-MreB (Figures 4E–H), with comparable diffusion constants of two detectable fractions, with the main difference that 63% of molecules rather than 54% of YFP-MreB molecules were in the slow-mobility/filament bound fraction (Figures 4E,H), supporting the view that we were able to realistically track MreB molecules in live cells.

### RodA and PbpH Single Molecule Dynamics Suggest Slow-Moving, Synthetically Active Molecules and Freely Diffusive Molecules

Next we wanted to compare the single molecule behavior of MreB with two proteins involved in the peptidoglycan synthesis machinery, RodA and PbpH. We expressed both fusions from the amylase locus using very low inducer conditions, such that very few copies were expressed in addition to wild type RodA or PbpH. In both cases, by applying a GMM curve fit, we could observe that the data can be well described by two mobile populations (RodA: *R*^2^ = 0.9129, PbpH: *R*^2^ = 0.8826) (Figures 4B–D, goodness of fit analysis: Supplementary Figure S4E). Assuming a single population did not fit with the clearly visible presence of static and mobile molecules seen in SMT acquisitions (see Supplementary Movie S3 as an example for YFP-PbpH), and with the non-Gaussian distribution of steps (Figures 4B,C). These data suggest that about 40% of RodA molecules are in a slow-moving (0.099 ± 0.008 μm^2^s^–1^) mode, and 60% in a fast-mobile mode (0.613 ± 0.08 μm^2^s^–1^, Supplementary Figure S5B), while 55% of PbpH molecules are engaged in low mobility, and thus possibly in cell wall synthesis (0.08 ± 0.005 μm^2^s^–1^) and 45% in a mobile mode (0.66 ± 0.09 μm^2^s^–1^, Supplementary Figure S5B). The diffusion constants determined for freely diffusing, single span membrane proteins is in the same range as those determined for RodA and PbpH (note that diffusion constants of membrane proteins depend on the number of transmembrane spans rather than molecule size, and is not much lower than that of cytosolic proteins) (Lucena et al., 2018), so it is likely that the fast-mobile fractions correspond to freely diffusive molecules. Diffusion of RodA is somewhat abnormal as it contains 10 transmembrane spans, for which we have no explanation. These data suggest that similar to Pbp2a in *E. coli* (Lee et al., 2014), *B. subtilis* RodA and PbpH change between a bound and diffusive state within the membrane. Heat maps of RodA and of PbpH showed a more dispersed pattern than observed for MreB (Figure 4A), in agreement with the spot-like localization of RodA or PbpH versus the filamentous structures of MreB seen in TIRF-SIM (Supplementary Figure S2).

Please note that the fast acquisition rate we chose does not allow us to discriminate between slow-moving and static molecules. For Pbph2 from *E. coli*, it has recently been shown that three populations exist, a diffusive, a slow-mobile and a static one (Ozbaykal et al., 2020). The purpose of our study was to compare dynamics of MreB, RodA and PbpH, we therefore did not track all proteins with many different acquisition rates, but chose one that allowed us to best distinguish between diffusive and slow mobile molecules, assuming the latter to be associated with cell wall extension or repair. Moreover, we did not find a large degree of directed motion for the slow mobile/static fractions we determined for MreB, RodA, and PbpH, which would have been predicted from the directed motion of MreB filaments. To investigate this point, we analyzed GMM fitting that we employed for comparison of changes over differing conditions, which assumes a Brownian diffusion model (MSD: α = 1). We analyzed tracks that are sufficiently long to yield information on a possibly confined or directed movement of the respective molecule (MSD: α ≠ 1). As the average track length for the data sets was around seven steps, and because MSDs calculated from sets of short tracks intrinsically tend toward linearity, we firstly categorized the α-value of molecules showing at least nine steps via a classical non-linear least squares fit (*MSD*(*t*)∼4*D*_α_*t*^*a*^). For YFP-MreB, for example, this threshold left ∼20% of tracks for analysis. Supplementary Figures S4A–D shows that more than 90% of all MreB tracks are characterized by Brownian motion, while less than 10% show directed (alpha close to “2”) or constrained (alpha close to “0”) motion. Even very long tracks (18 steps or more) revealed less than 20% constrained or directed motion, showing that at the short lifetime of SMT molecules, a large majority of molecules behave in a Brownian manner. Analysis of RodA and PbpH alpha values yielded similar results (not shown). Thus, directed motion of MreB filaments can only be differentiated and characterized if longer time frames (seconds rather than milliseconds) are used, which must be considered when using SMT.

We also determined localization errors, calculated from the *y*-axis intercept of the single trajectory MSD curves, generated form the dataset by the software SMTracker (Rosch et al., 2018b). We obtained about 54 nm for MreB and RodA, or 50 nm for PbpH (Supplementary Figure S1F). To compare this to the localization error of a protein that is statically anchored in the cell, we determined the localization error for ComEB, which is part of a large cell wall-associated DNA uptake machinery, during the state of competence (Kaufenstein et al., 2011). ComEB is a soluble part of this membrane-spanning multiprotein complex, and we found a localization error of 39 nm for ComEB-YFP (Supplementary Figure S1F). Thus, there is higher motion for MreB, RodA, and PbpH compared to a static molecule, likely because even the slow-mobile population shows movement relative to the cell wall.

Taken together, these experiments show that single molecule tracking can distinguish between two mobility modes for RodA and PbpH, and support the idea that freely diffusing RodA and PbpH molecules are recruited to sites of active cell wall synthesis, and that about half of the molecules are engaged in a transient PGEM complex (or other activities leading to constrained mobility, Vigouroux et al., 2020), and the other half are in a diffusive state in search for sites of active cell wall synthesis.

### Osmotic Stress Mainly Influences the Dynamics of MreB, but Not of RodA and PbpH

After we observed that diffusion of MreB, RodA, and PbpH was distinguishable during normal growth, we investigated if and how diffusion would be influenced under non-optimal growth conditions. Since peptidoglycan is important to counteract osmotic stress we analyzed how proteins involved in PG synthesis behave at a single molecule level under different osmotic stress conditions. We grew the cells in minimal media (S_750_) until exponential phase (OD: 0.4–0.6) and added 0.5 M NaCl (osmotic and ionic stress) or 1 M Sorbitol (osmotic stress) to the media for 30 min before mounting cells on agar pads containing normal growth medium (lacking stressors) and microscopic acquisition. Both 0.5 M NaCl and 1 M Sorbitol apply moderate stress to the cells (Hoffmann et al., 2013). Note that there was no detectable effect on cell shape in cells expressing a low amount of YFP-MreB as additional copy, compared with completely wild type cells (Supplementary Figure S1D), and cells continued to grow, albeit somewhat more slowly (Supplementary Figure S1B).

**FIGURE 5:**
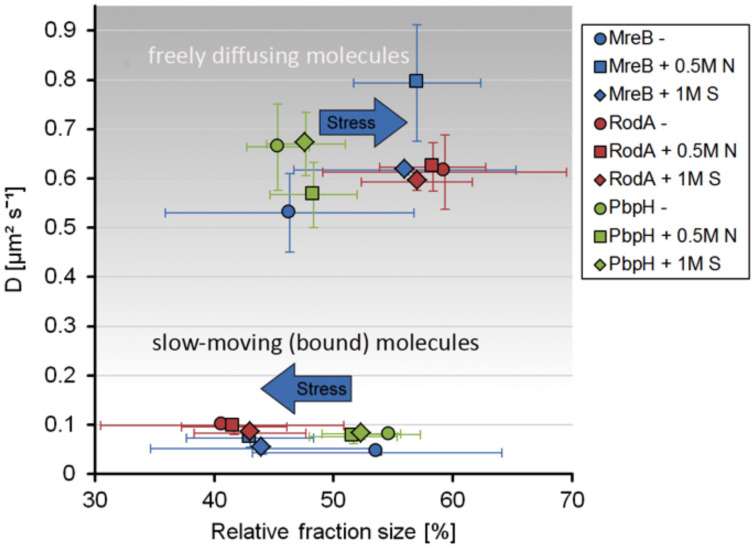
Scatterplot of the relative fraction sizes and diffusion D [μm^2^s^– 1^] as identified by non-simultaneous GMM fit for MreB, PbpH, and RodA under normal growth and with the addition of 0.5 M NaCl or 1 M Sorbitol.

Strikingly, the three proteins reacted to osmotic stress in a different manner. The slow-moving MreB population increased in its diffusion constant (0.074 μm^2^s^–1^ ± 0.02) and was reduced to 43% ± 5.3 after addition of NaCl, and diffusion constants of the faster population increased after salt (0.79 μm^2^s^–1^ ± 0.12) and sorbitol (0.62 μm^2^s^–1^ ± 0.009) stress, while the diffusion pattern of PbpH did not change considerably in response to osmotic stress (Figure 5). Likewise, the slower RodA population did not exhibit a major change in the diffusion constant after salt stress (0.096 μm^2^s^–1^ ± 0.016), but a minor decrease in response to sorbitol (0.084 μm^2^s^–1^ ± 0.01) (Figure 5 and Supplementary Figure S5A). Similar to the unstressed conditions, two populations with distinct diffusion constants for RodA and PbpH and MreB respectively were sufficient to explain the spread of probabilities; in all cases, *R*^2^ tests showed highly reliable data qualities (Figure 5 and Supplementary Figures S4E, S5B). For the three replicates done on three separate days that are summarized in Figure 5, the change in dynamics under both stress conditions was significantly different (α = 0.01) from the respective unstressed condition for MreB and for RodA, but not for PbpH (Supplementary Figures S5A,B). Even though statistically significant for RodA, the difference in dynamics is minimal compared to that of MreB.

A similar effect of stress response on MreB dynamics was observed for tracking of a GFP-Mbl fusion, driven by the original promotor, as sole source of the protein in the cell (Dominguez-Escobar et al., 2011), in that the fast population increased under osmotic stress (Figures 4E–H), suggesting that similar to MreB, Mbl changed from a more polymerized to a less polymerized ratio after stress induction.

Taken together these findings suggest that while MreB shows a considerable response to osmotic stress, RodA and PbpH seem to be not or only marginally affected in their presumed synthesis-engaged mode.

### MreB Localization and the Pattern of Peptidoglycan Synthesis Change Under Osmotic Stress

Because we observed that osmotic stress conditions have a different effect on the diffusion of MreB and of RodA and PbpH being involved in PG-synthesis, we further investigated if the patterns of PG-synthesis and of MreB macrostructures are also altered under these conditions. We used the fluorescent-D-amino acid stain HADA to visualize the insertion of peptidoglycan under normal growth and after the addition of 1 M Sorbitol or 0.5 M NaCl.

**FIGURE 6:**
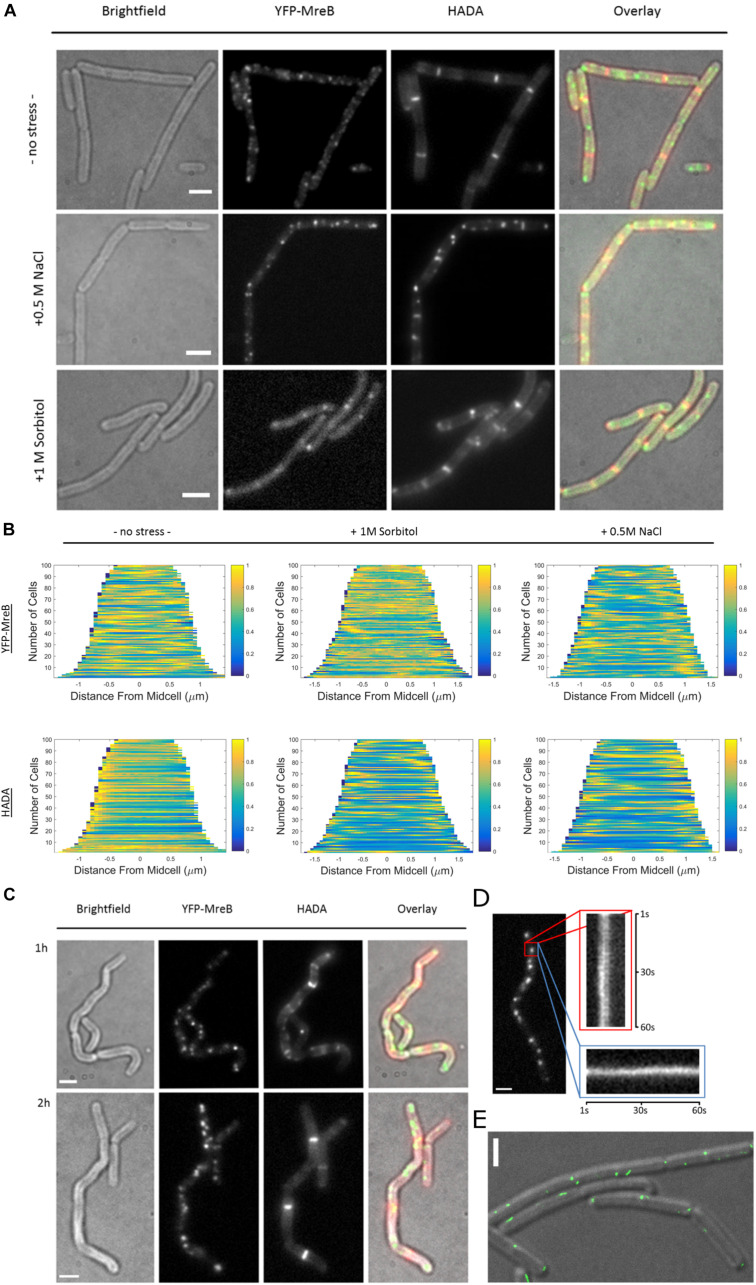
Fluorescent D-amino acid stain reveals MreB localization and the pattern of peptidoglycan synthesis change under osmotic stress. **(A)** Bright field, YFP-MreB (green-channel), HADA (red-channel, 0.5 mM) and overlay images of *B. subtilis* cells in exponential phase, expressing YFP-MreB under the control of the xylose promotor (+0.01% xylose) in S750 media: 20 min HADA staining without stress and with added 0.5 M NaCl or 1 M Sorbitol, images taken after washing 3 times with PBS, scalebar 2 μm; **(B)** Demograph of the distribution of YFP-MreB and HADA signal throughout *n* = 100 cells during normal growth or with added 0.5 M NaCl or 1; **(C)** Brightfield, YFP-MreB (green-channel), HADA (red-channel, 0.5 mM) and overlay images of *B. subtilis* expressing YFP-MreB under the control of the xylose promotor (+0.01% Xyl) in S750 media: 20 min HADA staining with added 0.5 M NaCl after 1 and 2 h, images taken after washing three times with PBS, scalebar 2 μm; **(D)** Line-kymograph of the movement of an exemplary YFP-MreB macrostructure (red rectangle) 2 h after 0.5 M NaCl addition in *x*- (red); *y*-direction (blue) over 60 s; **(E)** Gated-STED image of YFP-MreB after 2 h of 0.5M NaCl addition (green), overlaid on a DIC image of the cells; scale bars 2 μm.

When cells were grown to exponential phase in minimal media (S_750_) and then stained for 20 min with HADA, we observed a similar PG-pattern to what was previously described for *B. subtilis* by other groups (Kuru et al., 2012; Hsu et al., 2017), a weak staining of the lateral cell wall and strong staining of the septum (Figure 6A). Interestingly, after inducing osmotic stress, this pattern changed dramatically, and HADA staining became discontinuous and somewhat “patchy,” especially during salt stress. Similarly, the pattern of localization for MreB changed from more dispersed filaments and spots toward fewer and brighter structures (Figure 6A). These changes corroborate with the SMT data showing a reduction in the static fraction of MreB (Figure 5). Changes in the HADA and YFP-MreB patterns can be seen in the demographs, in which the positioning of signals is scored relative to the cell center (Figure 6B). Importantly, cells continued to grow after stress induction (Supplementary Figure S1B), at a slightly lower rate than before stress induction, in agreement with the marginal changes in the slow/static fractions of RodA and of PbpH (Supplementary Figure S5A and Figure 5). Both enzymes apparently continue to synthesize the cell wall with little reduction of the fraction of enzymes actively involved in cell wall extension.

### Fewer and Less Mobile MreB Filament Assemblies Form During Osmotic Stress While the Normal PG-Synthesis Pattern Recovers

Because of the distinct changes in the PG-synthesis pattern and in MreB localization dynamics shortly after induction of osmotic stress, we continued to monitor these patterns over time. After 1 h the cells became markedly bent and crooked (Figure 6C). Using HADA-staining we observed the synthesis of PG over the prior 20 min respectively; cells continued to grow, indicated by the incorporation of HADA, even though the pattern of PG-synthesis was discontinuous and spotty (Figure 6C and Supplementary Figure S6). MreB macrostructures localized mainly in short, immobile assemblies (Figure 6C), in a similar manner as observed 20 min after induction of osmotic stress (Figure 6A). Strikingly, after 2 h, cells appeared to straighten out, and the pattern of PG-synthesis had recovered to a more uniform state (Figure 6C), similar to what was previously observed for non-stressed cells. In contrast, MreB localization remained similar to post-stress conditions, with fewer and brighter structures (Figures 6A,C). These macrostructures were largely immobile, or very slow moving, during epifluorescence microscopic acquisitions (Figure 6D). To visualize YFP-MreB filaments with highest resolution available to us, we used G-STED (where sub-50 nm in x/y direction can be reached), which revealed that a substantial number of these structures corresponded to short, misaligned filamentous assemblies (Figure 6E). Strikingly, some structures formed by MreB during osmotic stress ran in parallel to the long axis, and not perpendicular, as under optimal growth conditions. In some cases we could observe very long filaments (several hundred nanometers), stretching along the long axis of the cell (Supplementary Figure S6). Note that STED will not visualize short (weakly fluorescent) mobile MreB filaments, which may well be present under these conditions (200 Hz unidirectional scanning, four-line averaging). Given that the pattern of cell wall synthesis recovered during that time, it is clear that this can occur in spite of the presence of more stationary MreB filaments. The same behavior was observed for a functional GFP-Mbl fusion expressed from the original gene locus under control of its original promoter (data not shown).

**FIGURE 7:**
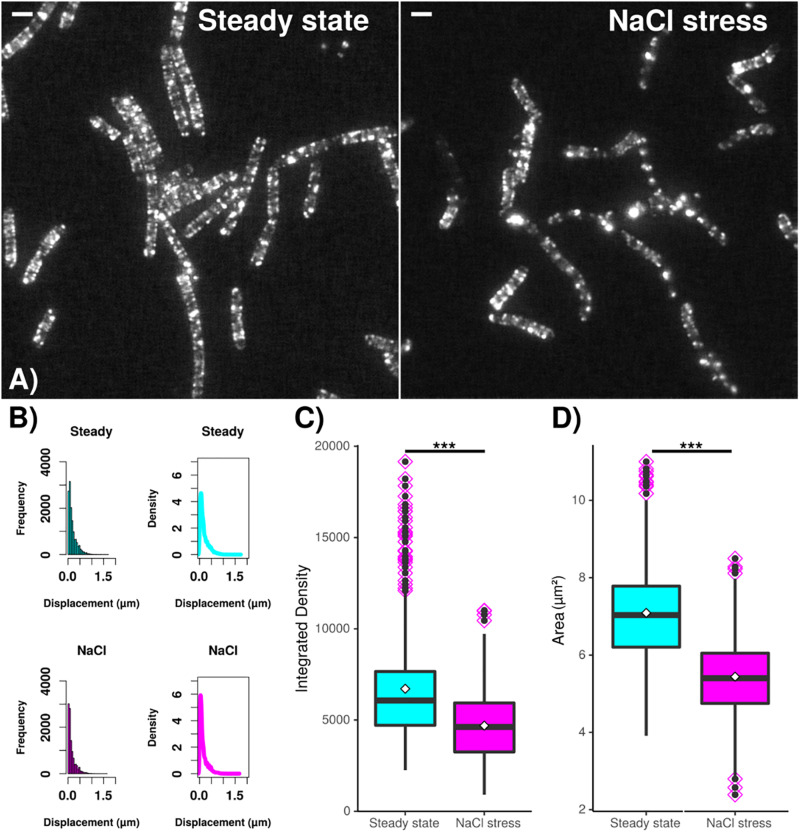
Dynamics and assembly/maintenance of *B. subtilis* MreB are affected by salt stress. SIM time lapse experiments. **(A)** Field of *B. subtilis* cells expressing GFP-MreB (from the ectopic *amy*-locus) during exponential growth (“steady state”) and 30 min after salt stress (“NaCl”). White bars 2 μm. **(B)** Frequency and density of displacements, indicating filament speed. **(C)** Box plots for integrated density and **(D)** area of fluorescence, indicating the extent of filament formation for GFP-MreB. Black horizontal lines indicate means, dots in pink diamonds indicate outliers. Data from *n* = 481 (steady state) or *n* = 471 (NaCl) cells. ***indicates *p* value of less than 0.001.

In order to verify changes in MreB filaments following osmotic stress, we tracked the movement of GFP-MreB filaments using SIM tracking. Figure 7A shows that the localization pattern of GFP-MreB changed after addition of sodium chloride and became more patchy. Analyzing a similar number of cells for both conditions (*n* = 471 steady state, *n* = 481 salt stress), we found that the displacement was decreased toward lower step sizes (Figure 7B) (median 124 nm steady state to 93 nm after stress), indicating that filament motion became lower, and that both, integrated density of filaments (mean 6713 AU steady state to 4694 AU after salt stress) and area occupied by filaments (mean 7.09 μm^2^ steady state to 5.44 μm^2^ after stress) became significantly smaller (Figure 7C). These data support the SMT analyses in that less MreB is associated with filamentous structures, and that more molecules become diffusive. Addition of sorbitol yielded very similar shifts in displacement, integrated density and area of GFP-MreB filaments (data not shown).

We also observed changes in the MreB localization patterns during other stress conditions using STED. After 1 h of incubation without shaking (no aeration) there were no longer filaments visible but mostly small immobile spots (Supplementary Figure S6C). After 5 min of 50°C heat shock we saw a pattern of shorter, seemingly more disorganized filaments (Supplementary Figure S6E), revealing that MreB filaments strongly react to different stress conditions.

Taken together, our findings reveal that changing environmental conditions have a significant effect on both the localization and dynamics of MreB filaments and of diffusing MreB molecules, and that regular incorporation of new cell wall material and regular dynamics of RodA and PbpH after stress adaptation is incongruent with MreB mobility.

## Discussion

The question how large protein machineries assemble and orchestrate synthetic activity is of broad biological interest. Of special interest is the question how the multitude of transglycosylases (TG), transpeptidases (TP) and hydrolases that are involved in cell growth through extension of the cell wall is coordinated such that their activity is distributed along a cylindrical surface, in case of rod shaped bacteria. However, in spite of the pivotal role of the bacterial cell wall as a major target for antibiotics, and despite decades of research, the 3D structure of the cell wall and its mode of synthesis in terms of spatiotemporal enzyme coordination are still poorly understood. We have used a combination of super-resolution fluorescence microscopy (SIM and STED) and single molecule tracking (SMT) to investigate the localization and dynamics of two cell wall synthesis enzymes and the equally important MreB protein at high temporal and spatial resolution.

According to an earlier model, peptidoglycan-extending (PGE) enzymes set up a stable complex, the PGE machinery (PGEM). A tight interaction and coordination of the extension of glycan strands (TPs), the crosslinking of these via peptide side chains (TPs) and the removal of hindering crosslinks (hydrolases) was assumed to be required for the cell wall growth, and to ensure that especially hydrolases do not act at unwanted places within the wall (Holtje, 1998). Several recent studies have shown that cell wall synthesis enzymes behave in a more intricate manner (Lee et al., 2014; Rohs et al., 2018; Vigouroux et al., 2020), and for *B. subtilis*, it has been shown that the activity of the so called Rod complex, containing RodA and associated class B PBPs, and of class A Pbps must be well balanced (possibly by MreB) to achieve correct cell morphology (Dion et al., 2019). In our work, we aimed at visualizing dynamics of RodA, PbpH (two components of the Rod complex) and MreB with the same imaging settings. Our findings argue against the existence of a multiprotein complex that is stable over many minutes and suggest that enzymes of the Rod complex in *Bacillus subtilis* act in a mode consisting of a mixture of diffusive motion and slow/static mobility, with MreB movement and filament formation changing during phases of growth adaptation.

We show by TIRF-SIM and verify that the motion of RodA (TG) and of PbpH (TP) generally follows that of tracks perpendicular to the long axis of the rod shaped cell, with frequent tilts away from a 90° angle; very similar to what has been described for MreB filaments (Reimold et al., 2013). However, trajectories of RodA molecules are considerably shorter than those of MreB, and those of PbpH are even shorter than RodA. The observed MreB filaments mainly moved smoothly at a constant velocity (without stops) over a time period ofδ*t* = 8 ± 3*s*(*trajectorylengthof* 470*nm*). If we assume that the filaments are pushed or pulled along with the inserted PG-material and that the insertion is done by a putative complex of cell wall-synthesizing enzymes (the PGEM) complex, an estimation of the assembly and disassembly dynamics of the PGEM complex can be done. The PGEM complex should disassemble with MreB $k_{off}^{MreB}>\frac{1}{\delta t}=\left(0.125\neq 0.47\right)Hz$. PbpH and RodA are thought to form an essential part of the PGEM complex (Holtje, 1998). Consequently, the unbinding rates of PbpH and RodA should be in the same order of magnitude as that of MreB, and trajectory lengths of PbpH and RodA should be as long as the trajectory lengths of MreB. However, PbpH has a mean trajectory time of 2.4 ± 1.1 s, therefore the mean off-binding rate is PbpH$k_{off}^{pbpH}>\left(0.42\neq 0.19\right)Hz$, while RodA with 3.8 ± 2.1 s has a mean off rate of 0.26 *Hz*. Thus, cell wall synthesis by these two enzymes occurs quite discontinuously, including different on and off rates at the putative PGEM complex. It follows that enzymes involved in cell wall synthesis including RodA and PbpH bind and unbind to sites of synthesis with different kinetics (Supplementary Figure S7) during non-perturbed growth.

What may be the advantage of a dynamic synthesis-machinery? We can envision that rapid exchange of redundantly operating TGs and TPs increases the reaction speed to changes in the environment of the cell, helping the cell wall synthesis machinery to quickly adapt to e.g., presence of antibiotic compounds or changes in osmolarity. Secondly, based on pure speculation, it may be beneficial to synthesize many relatively short, interconnected PG strands rather than fewer, long strands, in order to increase cell wall flexibility. Thirdly, as synthesis of PG strands appears to occur in both directions, based on RodA and MreB movement (Reimold et al., 2013; Billaudeau et al., 2017) (this work), this mode may benefit from rapid on/off rates in case of crossing of strands: MreB filaments block another when their path crosses (Olshausen et al., 2013), but at a longer time scale, MreB filaments can cross the former path of a filament (Hussain et al., 2018), suggesting that PG strand orientation is not completely aligned and that collision of PG extension enzymes can occur.

A second key finding of our work is derived from SMT experiments, which show that RodA and PbpH change between slow and fast movement, such that about half of the existing molecules are in a slow mode (i.e., involved in PG synthesis) and half are freely diffusing. This is similar to what was described for Pbp2a in *E. coli*, a sister enzyme of PbpH, and shows that alike *E. coli* TP PBPs (Lee et al., 2014), *B. subtilis* PbpH and RodA act via diffusive motion and dynamic association with the rest of the elongation machinery. Of note, recent work has shown that Pbp2, a component of the *E. coli* Rod complex, shows three distinct mobility fractions, one freely diffusive, one slow mobile, and one entirely static (Ozbaykal et al., 2020). From our analyses, we can not distinguish if the slow mobile fraction we have observed for PbpH, RodA and MreB consist of two distinguishable fractions in analogy to Pbp2 from *E. coli*. For the purpose of comparing dynamics within the Rod complex, we have chosen to assume free diffusion for molecules not involved in active cell wall synthesis, and slow motion for enzymes associated with activities in synthesis, or filament formation in case of MreB.

It had been speculated that *B. subtilis* and *E. coli* extend their cell wall in different modes, i.e., predominant directed movement within a stable complex (Dominguez-Escobar et al., 2011; Garner et al., 2011) versus diffusive motion and dynamic association (Lee et al., 2014), respectively (Banzhaf and Typas, 2014). Thus, a picture emerges with the PGEM complex moving in a directed manner, together with MreB, being composed of dynamically associating and dissociating subunits that otherwise freely diffuse in the membrane, and MreB filaments possibly recruiting new monomers from freely diffusive and membrane-associated fractions.

An interesting finding of this work is that the mode of correlated movement of MreB, RodA and PbpH changed markedly when cells were challenged with osmotic stress. While the movement of PbpH and RodA was hardly affected, MreB showed a transient response to the stress condition, in that it became much more diffusive. The slow-moving fraction became smaller, and the freely diffusive fraction increased accordingly, while dynamics generally increased under moderate osmotic stress. The change was more pronounced in response to ionic stress than to mere osmotic stress. This increase in diffusing molecules to the expense of static molecules, visible at the single-molecule level, leads us to hypothesize that a fraction of MreB depolymerizes. In agreement with this idea we found that on a larger time-scale, the number of MreB macrostructures was considerably reduced, and the fewer visible MreB filaments became less mobile, some even completely immobile, for a transient period of about 2 h. During this time, the pattern of cell wall synthesis changed from a relatively uniform lateral insertion mode to a highly spotty, seemingly less coordinated mode (about 1 h after stress induction), while the cells continued to grow at a slightly reduced rate. Accordingly, cells became more bend and lost straight growth for 60–120 min. After 2 h, the pattern of cell wall synthesis developed back toward the more uniform pattern during non-perturbed growth, while MreB filaments still remained relatively immobile. During the whole time of the stress response, cell extension continued, albeit at slightly lower level, revealing that the wall extension machinery can continue its activity in spite of altered MreB filament numbers and decreased mobility. SR microscopy also showed that during stress adaptation, filament orientation can strongly deviate from the perpendicular pattern seen during non-perturbed growth (Dominguez-Escobar et al., 2011; Garner et al., 2011; van Teeffelen et al., 2011; Olshausen et al., 2013; Reimold et al., 2013). These data show that the activity of the cell wall synthesis machinery does not linearly scale with MreB filament dynamics, and is relatively robust against osmotic stress. However, cell wall synthesis was visibly less well organized in the absence of MreB movement, as cells showed abnormal cell morphology during the stress adaptation phase. Thus, while cell wall extension appears to be able to continue without the help of MreB rotation, optimal 3D arrangement of extension seems to require coordinated motion with MreB filaments.

Our findings support the idea that MreB optimizes the coordination of enzymes of the dynamic PGE machinery during rapid growth (Dion et al., 2019), and either confers a different role during stress adaptation, may be related to a regulatory role between cytosolic and membrane proteins, or needs to change its dynamics in order to keep a balance between the Rod complex and class A PBPs. Interestingly, MreB has been shown to functionally interact with translation elongation factor EF-Tu, in a 1:1 manner (Defeu Soufo et al., 2010, 2015). A strong shift from filament-bound to diffusive MreB following after, e.g., salt stress as observed by SMT might have an effect on translation, and/or vice-versa. The idea of a regulatory function of MreB is in agreement with findings that an *mreB* deletion can be suppressed by transcription factors involved in cell envelope stress response (Schirner and Errington, 2009). It may also explain why an *mreB* deletion can be rescued by increased magnesium concentrations (Formstone and Errington, 2005), which play an important role in many processes including cell wall synthesis. While our results are compatible with recent ideas of MreB establishing a platform to guide cell wall extension into a tubular mode (Hussain et al., 2018), they indicate that a second important function possibly lies in processes related to growth adaptation, as judged from its strongly altered pattern and dynamics during an adaptation phase.

It is striking to note that *B. subtilis* can grow as irregularly shaped/ovoid cells, e.g., in the absence of cell wall teichoic acids (Hussain et al., 2018). Therefore, rod cell shape *per se* is not a requirement for viability and efficient growth, while the presence of MreB paralogs is required, unless extragenic suppressors arise. These findings suggest that besides a putative role as coordinator between dynamically interacting components of the PGEM complex, MreB may perform an additional function during growth adaptation.

## Materials and Methods

### Growth Conditions

For all experiments, cells were inoculated from overnight culture in S7_50_ minimal media (fructose as carbon-source) with the respectively appropriate antibiotics (100 mg/ml ampicillin, 100 mg/ml spectinomycin, 5 mg/ml chloramphenicol) at 30°C shaking (200 rpm) and grown to exponential phase (OD ∼ 0.6). Expression of genes under the control of the *xyl*-promotor was induced by addition of xylose to a final concentration of 0.01%. To induce osmotic stress, NaCl or sorbitol were added to a final concentration of 0.5 M or 1 M respectively and, if not stated otherwise, cells were imaged 30 min after induction. If not stated otherwise, 4 μl of exponentially growing cell culture (OD ∼ 0.6) were spotted on glass coverslips (Roth) and fixed with a S7_50_ agarose pad (1% v/w), mounted on the microscope and subsequently imaged.

### Construction of Strains

For single crossover integration, the first 500 bp of *rodA* or of *pbpH* were cloned into pHJDS (Defeu Soufo and Graumann, 2006) using *Eco*RI/*Apa*I restriction sites. The generated plasmids were integrated into the *B. subtilis* chromosome by single crossover integration. For ectopic expression, entire *rodA* or *pbpH* genes were closed into pSG1191 (Feucht and Lewis, 2001), using *Eco*RI/*Apa*I restriction sites. Generated plasmids were integrated into the *amyE* locus via double crossover recombination, which was verified using starch assays.

### Strains Employed for Microscopy

Several strains were taken from several previous studies (Table 2). We imaged a GFP-MreB fusion (for TIRF-SIM, Figures 1, 2) or a YFP-MreB fusion (for single molecule tracking, SMT, Figures 3–5, and for epifluorescence and for STED imaging, Figure 6) expressed from the *amyE* locus under low induction (0.01% xylose), or a GFP-Mbl fusion expressed from the original gene locus under control of the original *mbl* promoter. For TIRF-SIM, a YFP-PbpH fusion expressed under control of the xylose promoter at the original gene locus or a YFP-RodA fusion expressed under control of the xylose promoter at the original gene locus were imaged (0.1% xylose). For SMT, YFP-RodA expressed from the *amyE* site was imaged (0.01% xylose), for YFP-PbpH, expressed from the original locus, the fusion was also induced at very low level (0.01% xylose, note that PbpH is non-essential). For SIM-tracking (Zeiss Elyra, Figure 7), GFP-MreB was used instead of YFP-MreB to achieve higher quantum efficiency using a fixed 488 nm laser line.

**TABLE 2:** List of strains.

Strain	Genotype	Resistance	Source
PY79	Wild type	–	Lab collection
JS36, YFP-MreB ectopic	*Pxyl-yfp−mreB::amyE*	Spectinomycin	Defeu Soufo and Graumann, 2006
JS12, GFP-MreB ectopic	*Pxyl-gfp−mreB::amyE*	Spectinomycin	Defeu Soufo and Graumann, 2006
GFP-Mbl original locus	*P_*mbl*_-gfp-mbl* (original locus)	Chloramphenicol	Dominguez-Escobar et al., 2011
YFP-RodA original locus	*Pxyl-yfp-rodA (original locus)*	Chloramphenicol	This work
YFP-RodA ectopic	*Pxyl-yfp-rodA::amyE*	Spectinomycin	This work
YFP-PbpH original locus	*P_*xyl*_-yfp-pbpH* (original locus)	Chloramphenicol	This work
YFP-PbpH ectopic	*Pxyl-yfp-pbpH::amyE*	Spectinomycin	This work

*B. subtilis fluorophore-fusion strains were derived from the wild type strain PY79.*

### Microscopy and Image Analysis

Slim field microscopy was performed on a customized Nikon Eclipse Ti microscope setup, (100 × oil-immersion objective, NA = 1.49), where central part of the 514 nm laser diode beam (100 mW max. power) was focused to the back focal plane of the objective. Fluorescent protein fusions were illuminated using a maximum of 160 W cm^–2^ on the image plane, to first bleach most of the fluorophores and subsequently track single molecules, which were identified as events with a single bleaching step. Due to low expression levels, single molecule levels were reached after 10 frames or earlier. A high-speed EMCCD camera (ImageEM X2, Hamamatsu) in frame transfer mode was used to acquire 20 ms stream acquisitions over 30 s.

Epifluorescence microscopy was performed using a Zeiss Axio Observer Z1 (100x oil-immersion, 1.46 NA, Photometrics Cascade II:512 camera). Gated-STED microscopy was performed using a Leica SP8 confocal microscope with a CW laser (200 hz, 4-line averages, unidirectional scanning). Demographs were generated with Oufti (Paintdakhi et al., 2016) from *n* = 100 cells each. Kymographs were generated with ImageJ^1^ using the MultipleKymograph plugin.

Single plane time lapse SIM imaging was done using 3 rotations; 5 phases; 16 bit, acquired by a ZEISS ELYRA PS.1 system (ANDOR Solis EMCCD (gain: 5, exposure time : 30 ms), 488 nm laser line with 200 mW (5%). ZEISS objective alpha Plan-Apochromat 100x/NA 1.46) setup. SIM reconstructions were processed using ZEN-Black software by ZEISS. ImageJ2/FIJI version 1.52p was used for processing and particle tracking (Rueden et al., 2017). Images were drift corrected using ImageStabilizer plugin. TrackMate plugin (v4.0.1) (Tinevez et al., 2017) was used to obtain Track Displacement values (D) using parameter settings blob diameter: 0.5 μm, threshold: 100–300, linking-maximum: 0.5 μm; gap-closure: 2. Results-tables are merged for each condition and analyzed by using workflows based on R-statistics and R-studio version 1.1.463^2^. D-frequencies were plotted as histograms using a set of *hist*()-expressions. Density plots were calculated using *density*()-function.

The custom built TIRF-SIM setup (Olshausen et al., 2013) was optimized for fast super-resolution imaging of dim, dynamic samples under a large field of view. Fast piezo mirrors (Physik Instrumente, Karlsruhe, Germany) quickly adjust illumination direction and grating phase, while a static segmented wave plate ensures the optimal polarization direction. An inverted DM-IRB microscope (Leica, Mannheim, Germany) with a NA = 1.46 Leica objective and a Hamamatsu Orca Flash 4.0 CMOS camera served as imaging unit. Prior to analysis all reconstructed time-lapse data were corrected for lateral drift using a custom written python code. Kymographs along the track of single filaments or elongated structures were created with ImageJ. The velocities of the protein of interest, their trajectory times and trajectory lengths were extracted from previously done kymographs. Trajectory angles were measured between the trajectory and the line between the poles of the rod-shaped bacterium.

### Single-Molecule Tracking

The obtained movies were cut to remove the initial bleaching phase. Single molecule tracks obtained via u-track (Jaqaman et al., 2008) and cell meshes generated with Oufti (Paintdakhi et al., 2016) were formatted and used in our custom software SMTracker (Rosch et al., 2018b) for analysis of single molecule behavior using SMMTrack (Schenk et al., 2017). Localization errors are determined by SMTracker according to Michalet (2010).

### Fluorescent D-Amino Acid (FDAA) Labeling

FDAA-labeling to visualize cell wall synthesis was performed utilizing HADA, according to Kuru et al. (2015). HADA was added to a final concentration of 0.5 mM, followed by incubation for 20 min shaking (30°C, 200 rpm, tubes were wrapped in aluminum foil to block out light) and subsequent washing three times with PBS, immediately before mounting and imaging.

## Data Availability Statement

All datasets generated for this study are included in the article/Supplementary Material.

## Author Contributions

SD performed the experiments shown in Figures 3–6 and Supplementary Figures S1, S4–S7, analyzed the data, and wrote the manuscript. JM performed the experiments in Figures 1, 2 and Supplementary Figures S2, S3, S7, and analyzed the data. LS performed the experiments on RodA and PbpH in Figures 3, 4 and analyzed the data. BM performed the experiments in Figure 7 and analyzed the data. JR contributed to Figures 1, 2 and Supplementary Figures S2, S3, and analyzed the data. PG and AR conceived of the study, analyzed the data, wrote the manuscript, and acquired the funding. All authors contributed to the article and approved the submitted version.

## Conflict of Interest

The authors declare that the research was conducted in the absence of any commercial or financial relationships that could be construed as a potential conflict of interest.

## Footnotes

1 http://imagej.net/Fiji

2 https://rstudio.com

## Supplementary Material

FIGURE S1 **(A)** Western blot of YFP-MreB expression. YFP-MreB, expressed in *B. subtilis* PY79, under xylose promotor control from the ectopic amy-locus, with addition of 1, 0.1, 0.01% xylose and without xylose respectively (Cells harvested at OD: 0.6, 1 h after induction with xylose) and Strep-YFP-MreB control, obtained via affinity chromatography after expression in *E. coli* BL21; **(B)** Growth-curve of *B. subtilis* PY79 in S750 media with and without addition of 0.5M NaCl, arrow indicates time point of addition of salt; **(C)** Epifluorescence images of cells expressing YFP-MreB from the ectopic amy locus under xylose promotor control (see **A**) with 0, 0.01, 0.1, and 1% xylose added respectively; **(D)** Morphology of wild type (“WT”) PY79 cells during exponential growth in minimal media (S750) +0.5M NaCl, and of cells expressing YFP-MreB from the ectopic *amy* locus under xylose promotor control (“YFP-MreB, +0.01% xylose); **(E)** Estimated localization error of sub-pixel events obtained from single molecule tracking of YFP-MreB, YFP-RodA, YFP-PbpH and YFP-ComEB (39 nm) expressed from *B. subtilis* PY79 in S7_50_ minimal media.

FIGURE S2 TIRF-SIM analysis of exponentially growing cells expressing GFP-MreB, YFP-RodA and YFP-PbpH from ectopic sites on the chromosome. **(A)** single acquisition of GFP-MreB expressed at low levels, **(B)** average intensity of 20 subsequent images (time projection) of MreB filament dynamics, **(C)** corresponding standard deviation (time projection) of MreB filaments. **(D)** example of a movie showing 4 frames of a GFP-MreB filament moving along a trajectory indicated by a green arrow, the dashed line indicates the right longitudinal border of the cell. **(E)** single acquisition of YFP-RodA expressed at low levels, **(F)** average intensity of 20 subsequent images (time projection) of YFP-RodA, **(G)** corresponding standard deviation (time projection), **(H)** single acquisition of YFP-PbpH expressed at low levels, **(I)** average intensity of 20 subsequent images (time projection) of YFP-PbpH, **(J)** corresponding standard deviation (time projection). **(K,L)** TIRF imaging showing stop and go events of PbpH and of RodA, taken at 1.25 Hz (800 ms exposures). Scale bar 0.5 μm. STD, standard deviation of time sequence, rightmost panels kymographs of movement, vertical scale bar 5 s.

FIGURE S3 Comparison of velocity and angle distribution of MreB, RodA, and PbpH On the right hand side the similar velocities of MreB, RodA, and PbpH can be seen. On the upper part of the figure the histograms of measured angles are shown. Standard deviations of the binned measurements are shown with error bars, the shaded area around the graphs is the error of the Gaussian fit, which is calculated with the square root of the value. MreB, RodA, and PbpH have very similar angles and velocities.

FIGURE S4 Alpha value distribution of YFP-MreB tracks for 9 step minimal track length cutoff **(A,B)** and 18 step minimal track length cutoff **(C,D)**, **(A,C)** Histogram of probability of alpha value skew, **(B,D)** scatter blot of distribution of alpha values vs. *D*_alpha_ with probability color map. **(E)** Goodness of fit for two population Gaussian mixture model of MreB, RodA, and PbpH under various growth conditions.

FIGURE S5 **(A)** Bubble plot of diffusion and fraction size for MreB, RodA, and PbpH under different stress conditions and **(B)** corresponding table. **(C)** Heat maps of the probabilistic distribution of tracks in a normalized cell for MreB, RodA, and PbpH after addition of 0.5 M NaCl or 1 M Sorbitol (dark red: higher probability, white: lower probability).

FIGURE S6 **(A)** Bright field, HADA (red-channel, 0.5 mM) and overlay images of *B. subtilis* PY79 cells in exponential phase in S750 media: 20 min HADA with added 0.5 M NaCl after 20 min, 1, 2 h; images taken after washing three times with PBS, scale bar 2 μm; **(B–E)** Gated-STED image of YFP-MreB under the control of the xylose promotor (+0.01% Xyl) in S750 media (green), overlaid on a DIC image of the cells after **(B)** exponentially growing cells, **(C)** 1 h of incubation without shaking, **(D)** 2 h of 0.5 M NaCl addition, **(E)** 5 min 50° heat shock, scale bars 2 μm.

FIGURE S7 Illustration of the measured trajectory lengths in the bacteria, PbpH (green) exchanges with the PG synthesis machinery significantly faster than RodA (blue), while both move less persistently than MreB (red).

MOVIE S1 TIRF-SIM time lapse of *Bacillus subtilis* cells expressing GFP-MreB from an ectopic site in the chromosome at low level induction, 2 images per second, movie speed 6 fps.

MOVIE S2 SMT stream acquisition of *B. subtilis* cells expressing YFP-MreB from an ectopic site in the chromosome at low level induction, 20 ms acquisitions (50 fps), movie speed 50 fps.

MOVIE S3 SMT stream acquisition of *B. subtilis* cells expressing YFP-PbpH from an ectopic site in the chromosome at low level induction, 20 ms acquisitions (50 fps), movie speed 50 fps.

## References

[B1] Banzhaf M. Typas A. (2014). Dynamic protein complexes for cell growth. *Proc. Natl. Acad. Sci. U.S.A.* 111 4355–4356. 10.1073/pnas.1402016111 24639518 PMC3970523

[B2] Billaudeau C. Chastanet A. Yao Z. Cornilleau C. Mirouze N. Fromion V. (2017). Contrasting mechanisms of growth in two model rod-shaped bacteria. *Nat. Commun.* 8:15370. 10.1038/ncomms15370 PMC5467245 28589952

[B3] Billaudeau C. Yao Z. Cornilleau C. Carballido-Lopez R. Chastanet A. (2019). MreB forms subdiffraction nanofilaments during active growth in *Bacillus subtilis*. *mBio* 10:e01879-18. 10.1128/mBio.01879-18 30696741 PMC6355991

[B4] Cabeen M. T. Jacobs-Wagner C. (2005). Bacterial cell shape. *Nat. Rev. Microbiol.* 3 601–610. 16012516 10.1038/nrmicro1205

[B5] Cho H. Wivagg C. N. Kapoor M. Barry Z. Rohs P. D. A. Suh H. (2016). Bacterial cell wall biogenesis is mediated by SEDS and PBP polymerase families functioning semi-autonomously. *Nat. Microbiol.* 1:16172. 10.1038/nmicrobiol.2016.172 PMC5030067 27643381

[B6] Defeu Soufo H. J. Graumann P. L. (2006). Dynamic localization and interaction with other *Bacillus subtilis* actin-like proteins are important for the function of MreB. *Mol. Microbiol.* 62 1340–1356. 10.1111/j.1365-2958.2006.05457.x 17064365

[B7] Defeu Soufo H. J. Reimold C. Breddermann H. Mannherz H. G. Graumann P. L. (2015). Translation elongation factor EF-Tu modulates filament formation of actin-like MreB protein *in vitro*. *J. Mol. Biol.* 427 1715–1727. 10.1016/j.jmb.2015.01.025 25676310

[B8] Defeu Soufo H. J. Reimold C. Linne U. Knust T. Gescher J. Graumann P. L. (2010). Bacterial translation elongation factor EF-Tu interacts and colocalizes with actin-like MreB protein. *Proc. Natl. Acad. Sci. U.S.A.* 107 3163–3168. 10.1073/pnas.0911979107 20133608 PMC2840354

[B9] Dion M. F. Kapoor M. Sun Y. Wilson S. Ryan J. Vigouroux A. (2019). *Bacillus subtilis* cell diameter is determined by the opposing actions of two distinct cell wall synthetic systems. *Nat. Microbiol.* 4 1294–1305. 10.1038/s41564-019-0439-0 31086310 PMC6656618

[B10] Dominguez-Escobar J. Chastanet A. Crevenna A. H. Fromion V. Wedlich-Soldner R. Carballido-Lopez R. (2011). Processive movement of MreB-associated cell wall biosynthetic complexes in bacteria. *Science* 333 225–228. 10.1126/science.1203466 21636744

[B11] El Najjar N. El Andari J. Kaimer C. Fritz G. Rosch T. C. Graumann P. L. (2018). Single-molecule tracking of DNA translocases in *Bacillus subtilis* reveals strikingly different dynamics of SftA, SpoIIIE, and FtsA. *Appl. Environ. Microbiol.* 84:e02610–02617. 10.1128/AEM.02610-17 29439991 PMC5881075

[B12] El Najjar N. Van Teeseling M. C. F. Mayer B. Hermann S. Thanbichler M. Graumann P. L. (2020). Bacterial cell growth is arrested by violet and blue, but not yellow light excitation during fluorescence microscopy. *BMC Mol. Cell. Biol.* 21:35. 10.1186/s12860-020-00277-y 32357828 PMC7193368

[B13] Emami K. Guyet A. Kawai Y. Devi J. Wu L. J. Allenby N. (2017). RodA as the missing glycosyltransferase in *Bacillus subtilis* and antibiotic discovery for the peptidoglycan polymerase pathway. *Nat. Microbiol.* 2:16253. 10.1038/nmicrobiol.2016.253 PMC5568705 28085152

[B14] Errington J. (2015). Bacterial morphogenesis and the enigmatic MreB helix. *Nat. Rev. Microbiol.* 13 241–248. 10.1038/nrmicro3398 25578957

[B15] Favini-Stabile S. Contreras-Martel C. Thielens N. Dessen A. (2013). MreB and MurG as scaffolds for the cytoplasmic steps of peptidoglycan biosynthesis. *Environ. Microbiol.* 15 3218–3228. 10.1111/1462-2920.12171 23826965

[B16] Feucht A. Lewis P. J. (2001). Improved plasmid vectors for the production of multiple fluorescent protein fusions in *Bacillus subtilis*. *Gene* 264 289–297. 10.1016/s0378-1119(01)00338-9 11250085

[B17] Figge R. M. Divakaruni A. V. Gober J. W. (2004). MreB, the cell shape-determining bacterial actin homologue, co-ordinates cell wall morphogenesis in *Caulobacter crescentus*. *Mol. Microbiol.* 51 1321–1332. 10.1111/j.1365-2958.2003.03936.x 14982627

[B18] Formstone A. Errington J. (2005). A magnesium-dependent *mreB* null mutant: implications for the role of *mreB* in *Bacillus subtilis*. *Mol. Microbiol.* 55 1646–1657. 10.1111/j.1365-2958.2005.04506.x 15752190

[B19] Garner E. C. Bernard R. Wang W. Zhuang X. Rudner D. Z. Mitchison T. (2011). Coupled, circumferential motions of the cell wall synthesis machinery and MreB filaments in *B. subtilis*. *Science* 333 222–225. 10.1126/science.1203285 21636745 PMC3235694

[B20] Graumann P. L. (2007). Cytoskeletal elements in bacteria. *Annu. Rev. Microbiol.* 61 589–618. 10.1146/annurev.micro.61.080706.093236 17506674

[B21] Hoffmann T. Wensing A. Brosius M. Steil L. Volker U. Bremer E. (2013). Osmotic control of opuA expression in *Bacillus subtilis* and its modulation in response to intracellular glycine betaine and proline pools. *J. Bacteriol.* 195 510–522. 10.1128/jb.01505-12 23175650 PMC3554007

[B22] Holtje J. V. (1998). Growth of the stress-bearing and shape-maintaining murein sacculus of *Escherichia coli*. *Microbiol. Mol. Biol. Rev.* 62 181–203. 10.1128/mmbr.62.1.181-203.1998 9529891 PMC98910

[B23] Hsu Y. P. Rittichier J. Kuru E. Yablonowski J. Pasciak E. Tekkam S. (2017). Full color palette of fluorescent d-amino acids for in situ labeling of bacterial cell walls. *Chem. Sci.* 8 6313–6321. 10.1039/c7sc01800b 28989665 PMC5628581

[B24] Hussain S. Wivagg C. N. Szwedziak P. Wong F. Schaefer K. Izore T. (2018). MreB filaments align along greatest principal membrane curvature to orient cell wall synthesis. *eLife* 7:e32471. 10.7554/eLife.32471 29469806 PMC5854468

[B25] Jaqaman K. Loerke D. Mettlen M. Kuwata H. Grinstein S. Schmid S. L. (2008). Robust single-particle tracking in live-cell time-lapse sequences. *Nat. Methods* 5 695–702. 10.1038/nmeth.1237 18641657 PMC2747604

[B26] Kaufenstein M. Van Der Laan M. Graumann P. L. (2011). The three-layered DNA uptake machinery at the cell pole in competent *Bacillus subtilis* cells is a stable complex. *J. Bacteriol.* 193 1633–1642. 10.1128/jb.01128-10 21278288 PMC3067657

[B27] Kawai Y. Daniel R. A. Errington J. (2009). Regulation of cell wall morphogenesis in *Bacillus subtilis* by recruitment of PBP1 to the MreB. *Mol. Microbiol.* 71 1131–1144. 10.1111/j.1365-2958.2009.06601.x 19192185

[B28] Kuru E. Hughes H. V. Brown P. J. Hall E. Tekkam S. Cava F. (2012). In Situ probing of newly synthesized peptidoglycan in live bacteria with fluorescent D-amino acids. *Angew. Chem. Int. Ed. Engl.* 51 12519–12523. 10.1002/anie.201206749 23055266 PMC3589519

[B29] Kuru E. Tekkam S. Hall E. Brun Y. V. Van Nieuwenhze M. S. (2015). Synthesis of fluorescent D-amino acids and their use for probing peptidoglycan synthesis and bacterial growth in situ. *Nat. Protoc.* 10 33–52. 10.1038/nprot.2014.197 25474031 PMC4300143

[B30] Lee T. K. Tropini C. Hsin J. Desmarais S. M. Ursell T. S. Gong E. (2014). A dynamically assembled cell wall synthesis machinery buffers cell growth. *Proc. Natl. Acad. Sci. U.S.A.* 111 4554–4559. 10.1073/pnas.1313826111 24550500 PMC3970539

[B31] Lucena D. Mauri M. Schmidt F. Eckhardt B. Graumann P. L. (2018). Microdomain formation is a general property of bacterial membrane proteins and induces heterogeneity of diffusion patterns. *BMC Biol.* 16:97. 10.1186/s12915-018-0561-0 30173665 PMC6120080

[B32] Meeske A. J. Riley E. P. Robins W. P. Uehara T. Mekalanos J. J. Kahne D. (2016). SEDS proteins are a widespread family of bacterial cell wall polymerases. *Nature* 537 634–638. 10.1038/nature19331 27525505 PMC5161649

[B33] Michalet X. (2010). Mean square displacement analysis of single-particle trajectories with localization error: Brownian motion in an isotropic medium. *Phys. Rev. E Stat. Nonlin. Soft Matter Phys.* 82:041914. 10.1103/PhysRevE.82.041914 PMC3055791 21230320

[B34] Mohammadi T. Karczmarek A. Crouvoisier M. Bouhss A. Mengin-Lecreulx D. Den Blaauwen T. (2007). The essential peptidoglycan glycosyltransferase MurG forms a complex with proteins involved in lateral envelope growth as well as with proteins involved in cell division in *Escherichia coli*. *Mol. Microbiol.* 65 1106–1121. 10.1111/j.1365-2958.2007.05851.x 17640276 PMC2170320

[B35] Morgenstein R. M. Bratton B. P. Nguyen J. P. Ouzounov N. Shaevitz J. W. Gitai Z. (2015). RodZ links MreB to cell wall synthesis to mediate MreB rotation and robust morphogenesis. *Proc. Natl. Acad. Sci. U.S.A.* 112 12510–12515. 10.1073/pnas.1509610112 26396257 PMC4603514

[B36] Olshausen P. V. Defeu Soufo H. J. Wicker K. Heintzmann R. Graumann P. L. Rohrbach A. (2013). Superresolution imaging of dynamic MreB filaments in *B. subtilis-*-a multiple-motor-driven transport? *Biophys. J.* 105 1171–1181. 10.1016/j.bpj.2013.07.038 24010660 PMC3762370

[B37] Ozbaykal G. Wollrab E. Simon F. Vigouroux A. Cordier B. Aristov A. (2020). The transpeptidase PBP2 governs initial localization and activity of the major cell-wall synthesis machinery in *E. coli*. *eLife* 9:e50629. 10.7554/eLife.50629 PMC7089770 32077853

[B38] Paintdakhi A. Parry B. Campos M. Irnov I. Elf J. Surovtsev I. (2016). Oufti: an integrated software package for high-accuracy, high-throughput quantitative microscopy analysis. *Mol. Microbiol.* 99 767–777. 10.1111/mmi.13264 26538279 PMC4752901

[B39] Reimold C. Defeu Soufo H. J. Dempwolff F. Graumann P. L. (2013). Motion of variable-length MreB filaments at the bacterial cell membrane influences cell morphology. *Mol. Biol. Cell* 24 2340–2349. 10.1091/mbc.e12-10-0728 23783036 PMC3727927

[B40] Rohs P. D. A. Buss J. Sim S. I. Squyres G. R. Srisuknimit V. Smith M. (2018). A central role for PBP2 in the activation of peptidoglycan polymerization by the bacterial cell elongation machinery. *PLoS Genet.* 14:e1007726. 10.1371/journal.pgen.1007726 30335755 PMC6207328

[B41] Rosch T. C. Altenburger S. Oviedo-Bocanegra L. Pediaditakis M. Najjar N. E. Fritz G. (2018a). Single molecule tracking reveals spatio-temporal dynamics of bacterial DNA repair centres. *Sci. Rep.* 8:16450. 10.1038/s41598-018-34572-8 PMC6219548 30401797

[B42] Rosch T. C. Oviedo-Bocanegra L. M. Fritz G. Graumann P. L. (2018b). SMTracker: a tool for quantitative analysis, exploration and visualization of single-molecule tracking data reveals highly dynamic binding of *B. subtilis* global repressor AbrB throughout the genome. *Sci. Rep.* 8:15747. 10.1038/s41598-018-33842-9 PMC6200787 30356068

[B43] Roth J. Mehl J. Rohrbach A. (2020). Fast TIRF-SIM imaging of dynamic, low-fluorescent biological samples. *Biomed. Optics Express* 11 4008– 4026. 10.1364/BOE.391561 PMC7510889 33014582

[B44] Rueden C. T. Schindelin J. Hiner M. C. Dezonia B. E. Walter A. E. Arena E. T. (2017). ImageJ2: ImageJ for the next generation of scientific image data. *BMC Bioinform.* 18:529. 10.1186/s12859-017-1934-z 29187165 PMC5708080

[B45] Salje J. Van Den Ent F. De Boer P. Lowe J. (2011). Direct membrane binding by bacterial actin MreB. *Mol. Cell* 43 478–487. 10.1016/j.molcel.2011.07.008 21816350 PMC3163269

[B46] Schenk K. Hervas A. B. Rosch T. C. Eisemann M. Schmitt B. A. Dahlke S. (2017). Rapid turnover of DnaA at replication origin regions contributes to initiation control of DNA replication. *PLoS Genet.* 13:e1006561. 10.1371/journal.pgen.1006561 28166228 PMC5319796

[B47] Schirner K. Errington J. (2009). The cell wall regulator {sigma}I specifically suppresses the lethal phenotype of *mbl* mutants in *Bacillus subtilis*. *J. Bacteriol.* 191 1404–1413. 10.1128/jb.01497-08 19114499 PMC2648184

[B48] Schirner K. Marles-Wright J. Lewis R. J. Errington J. (2009). Distinct and essential morphogenic functions for wall- and lipo-teichoic acids in *Bacillus subtilis*. *EMBO J.* 28 830–842. 10.1038/emboj.2009.25 19229300 PMC2670855

[B49] Tinevez J. Y. Perry N. Schindelin J. Hoopes G. M. Reynolds G. D. Laplantine E. (2017). TrackMate: an open and extensible platform for single-particle tracking. *Methods* 115 80–90. 10.1016/j.ymeth.2016.09.016 27713081

[B50] van Teeffelen S. Wang S. Furchtgott L. Huang K. C. Wingreen N. S. Shaevitz J. W. (2011). The bacterial actin MreB rotates, and rotation depends on cell-wall assembly. *Proc. Natl. Acad. Sci. U.S.A.* 108 15822–15827. 10.1073/pnas.1108999108 21903929 PMC3179079

[B51] Vigouroux A. Cordier B. Aristov A. Alvarez L. Ozbaykal G. Chaze T. (2020). Class-A penicillin binding proteins do not contribute to cell shape but repair cell-wall defects. *eLife* 9:e51998. 10.7554/eLife.51998 31904338 PMC7002073

[B52] Wei Y. Havasy T. Mcpherson D. C. Popham D. L. (2003). Rod shape determination by the *Bacillus subtilis* class B penicillin-binding proteins encoded by *pbpA* and *pbpH*. *J. Bacteriol.* 185 4717–4726. 10.1128/jb.185.16.4717-4726.2003 12896990 PMC166473

[B53] White C. L. Kitich A. Gober J. W. (2010). Positioning cell wall synthetic complexes by the bacterial morphogenetic proteins MreB and MreD. *Mol. Microbiol.* 76 616–633. 10.1111/j.1365-2958.2010.07108.x 20233306

[B54] Yang D. C. Blair K. M. Salama N. R. (2016). Staying in shape: the impact of cell shape on bacterial survival in diverse environments. *Microbiol. Mol. Biol. Rev.* 80 187–203. 10.1128/mmbr.00031-15 26864431 PMC4771367

