# Supplementary figures and images for: Super-Resolution Microscopy and Single-Molecule Tracking Reveal Distinct Adaptive Dynamics of MreB and of Cell Wall-Synthesis Enzymes
Source: Front Microbiol. 2020 Aug 20;11:1946. doi: 10.3389/fmicb.2020.01946 (PMC7468405; doi:10.3389/fmicb.2020.01946)

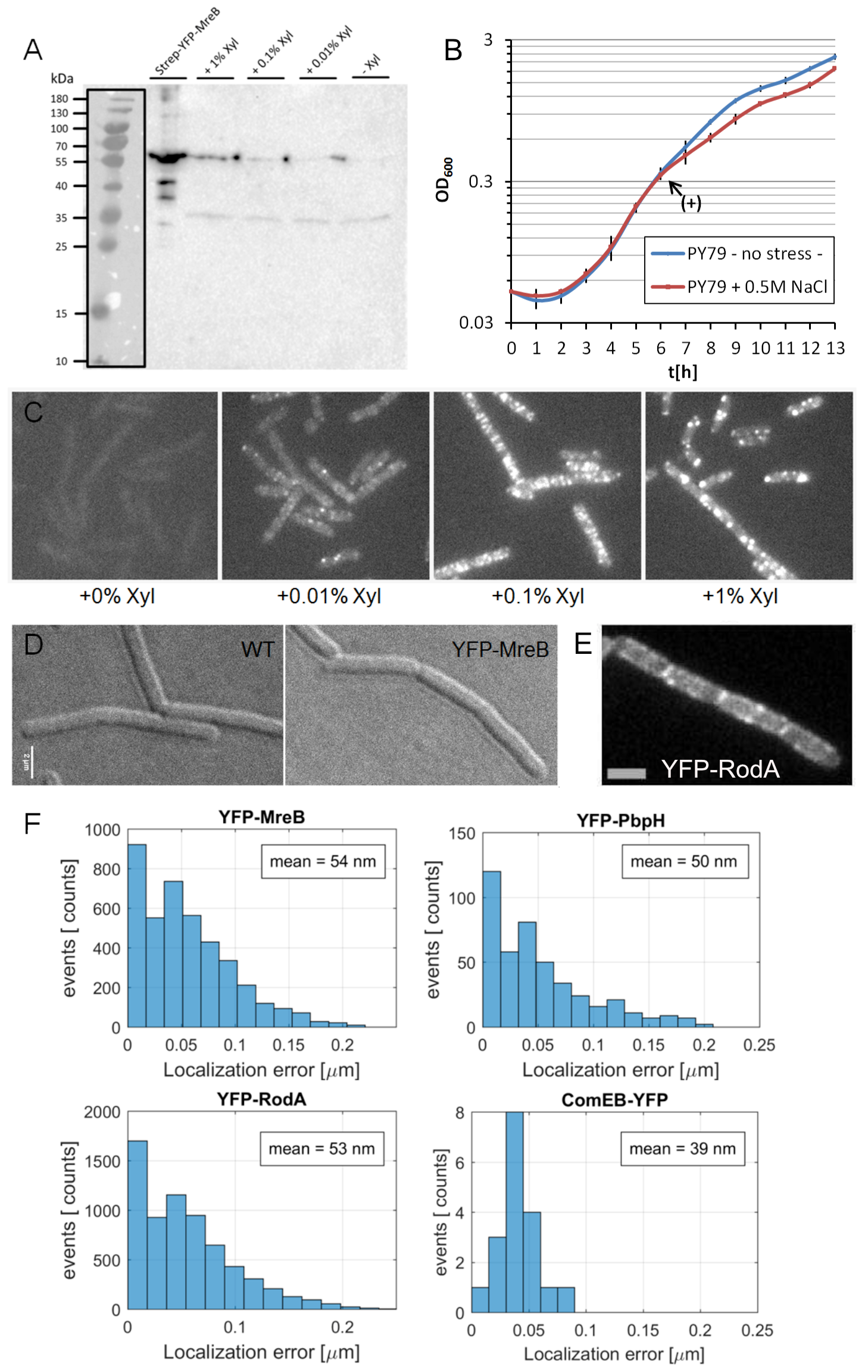

Supplement: FIGURE S1 — (A) Western blot of YFP-MreB expression. YFP-MreB, expressed in B. subtilis PY79, under xylose promotor control from the ectopic amy-locus, with addition of 1, 0.1, 0.01% xylose and without xylose respectively (Cells harvested at OD: 0.6, 1 h after induction with xylose) and Strep-YFP-MreB control, obtained via affinity chromatography after expression in E. coli BL21; (B) Growth-curve of B. subtilis PY79 in S750 media with and without addition of 0.5M NaCl, arrow indicates time point of addition of salt; (C) Epifluorescence images of cells expressing YFP-MreB from the ectopic amy locus under xylose promotor control (see A) with 0, 0.01, 0.1, and 1% xylose added respectively; (D) Morphology of wild type (“WT”) PY79 cells during exponential growth in minimal media (S750) +0.5M NaCl, and of cells expressing YFP-MreB from the ectopic amy locus under xylose promotor control (“YFP-MreB, +0.01% xylose); (E) Estimated localization error of sub-pixel events obtained from single molecule tracking of YFP-MreB, YFP-RodA, YFP-PbpH and YFP-ComEB (39 nm) expressed from B. subtilis PY79 in S750 minimal media. [file Image_1.TIFF]

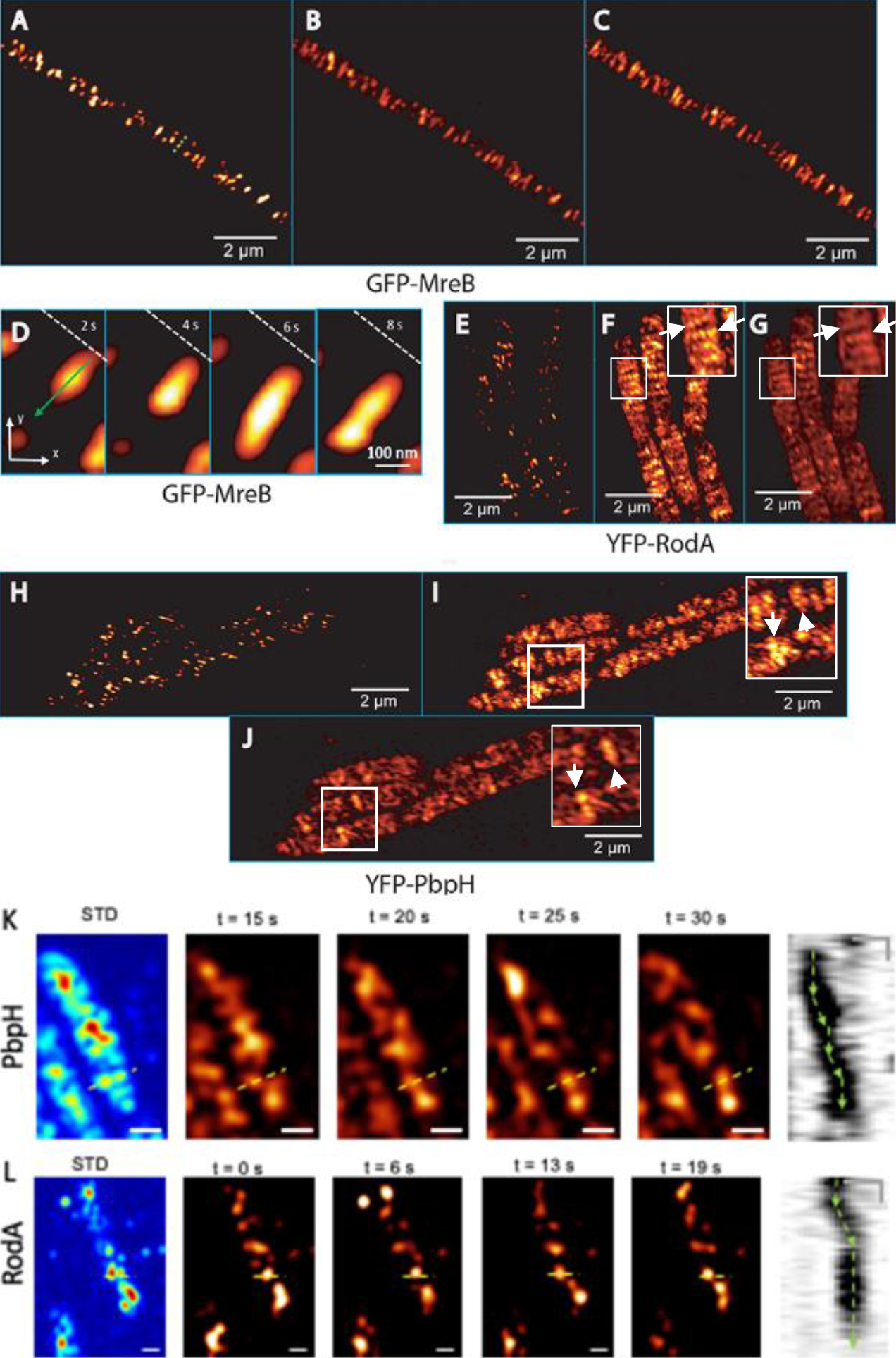

Supplement: FIGURE S2 — TIRF-SIM analysis of exponentially growing cells expressing GFP-MreB, YFP-RodA and YFP-PbpH from ectopic sites on the chromosome. (A) single acquisition of GFP-MreB expressed at low levels, (B) average intensity of 20 subsequent images (time projection) of MreB filament dynamics, (C) corresponding standard deviation (time projection) of MreB filaments. (D) example of a movie showing 4 frames of a GFP-MreB filament moving along a trajectory indicated by a green arrow, the dashed line indicates the right longitudinal border of the cell. (E) single acquisition of YFP-RodA expressed at low levels, (F) average intensity of 20 subsequent images (time projection) of YFP-RodA, (G) corresponding standard deviation (time projection), (H) single acquisition of YFP-PbpH expressed at low levels, (I) average intensity of 20 subsequent images (time projection) of YFP-PbpH, (J) corresponding standard deviation (time projection). (K,L) TIRF imaging showing stop and go events of PbpH and of RodA, taken at 1.25 Hz (800 ms exposures). Scale bar 0.5 μm. STD, standard deviation of time sequence, rightmost panels kymographs of movement, vertical scale bar 5 s. [file Image_2.tif]

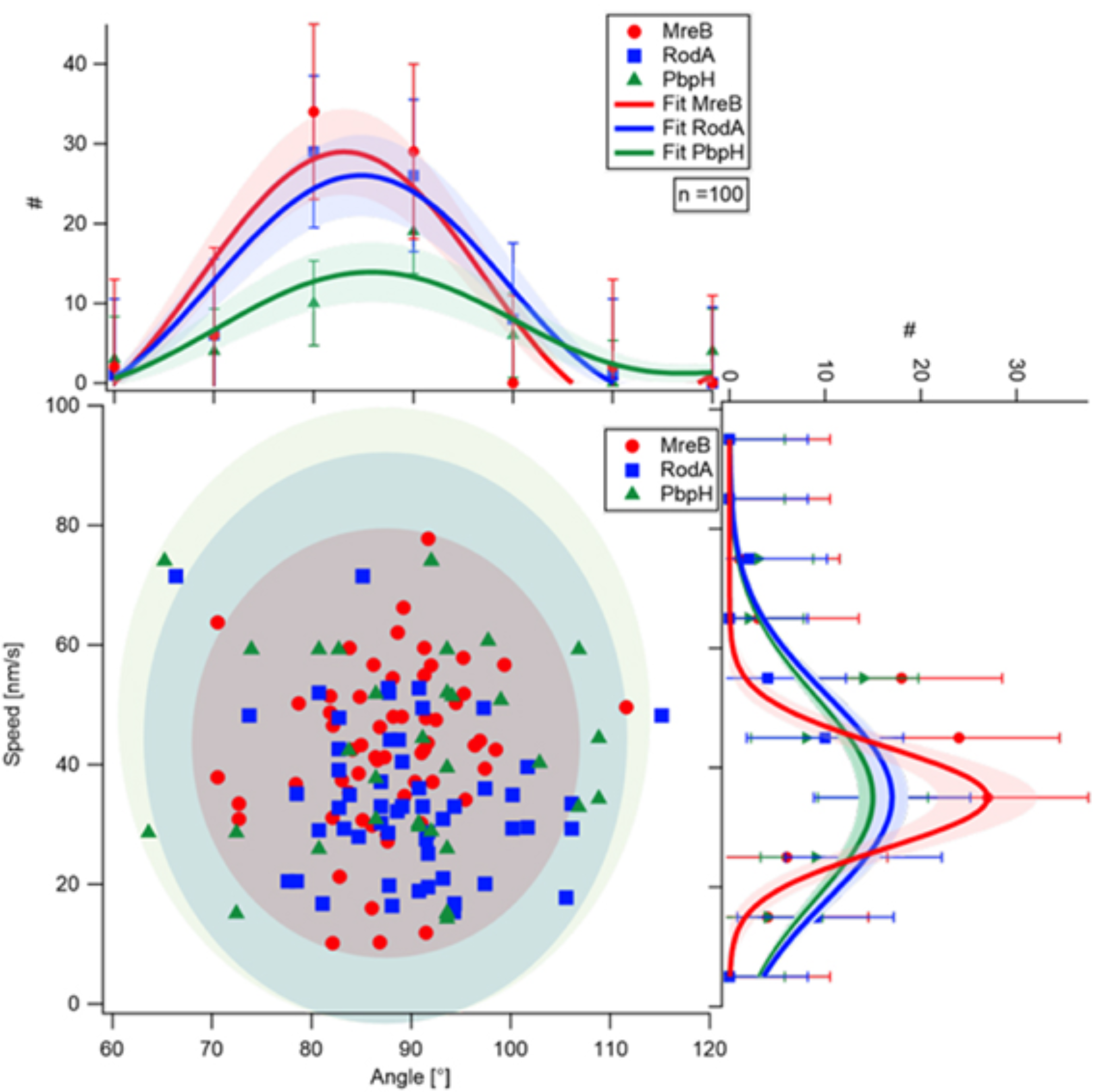

Supplement: FIGURE S3 — Comparison of velocity and angle distribution of MreB, RodA, and PbpH On the right hand side the similar velocities of MreB, RodA, and PbpH can be seen. On the upper part of the figure the histograms of measured angles are shown. Standard deviations of the binned measurements are shown with error bars, the shaded area around the graphs is the error of the Gaussian fit, which is calculated with the square root of the value. MreB, RodA, and PbpH have very similar angles and velocities. [file Image_3.TIF]

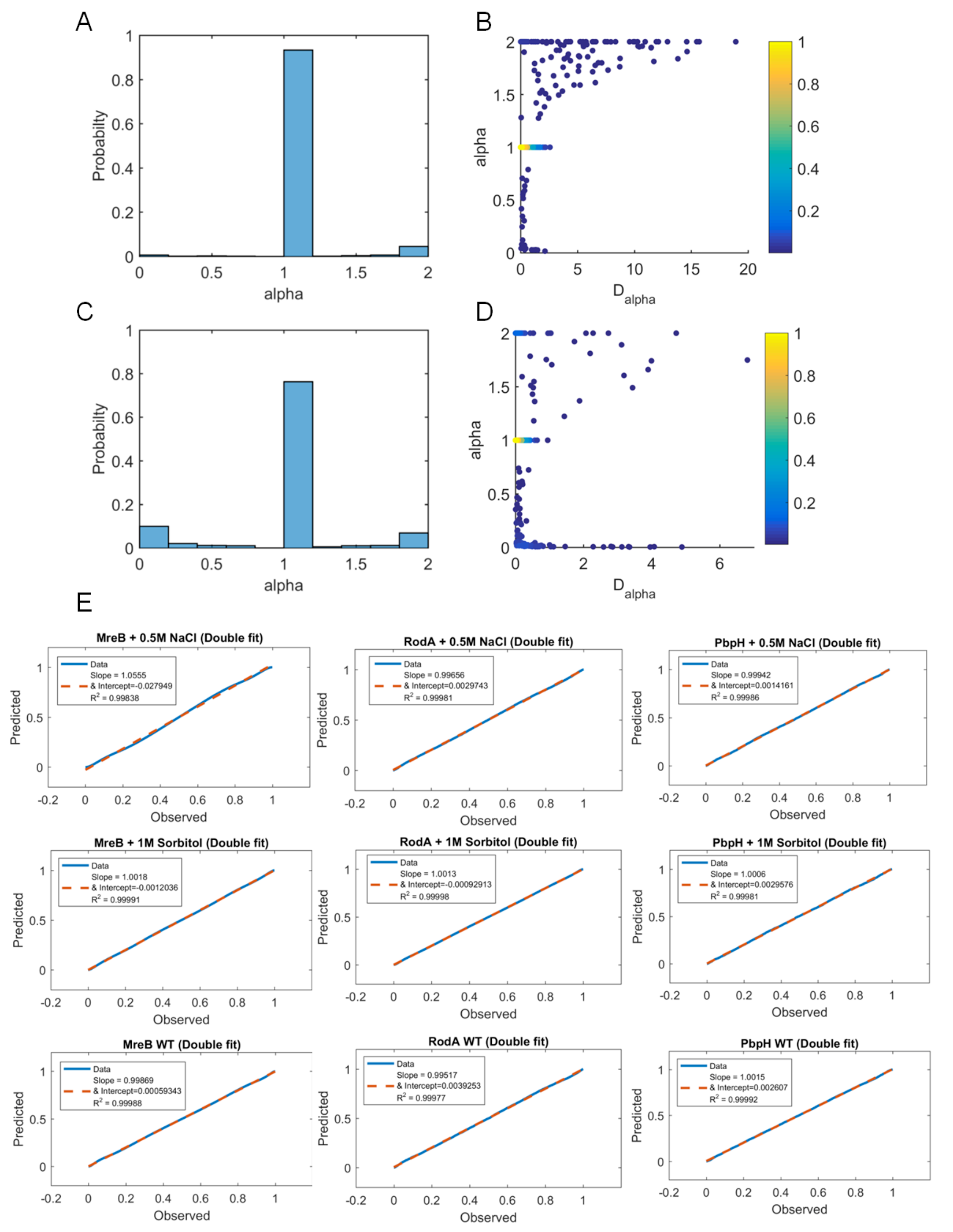

Supplement: FIGURE S4 — Alpha value distribution of YFP-MreB tracks for 9 step minimal track length cutoff (A,B) and 18 step minimal track length cutoff (C,D), (A,C) Histogram of probability of alpha value skew, (B,D) scatter blot of distribution of alpha values vs. Dalpha with probability color map. (E) Goodness of fit for two population Gaussian mixture model of MreB, RodA, and PbpH under various growth conditions. [file Image_4.TIF]

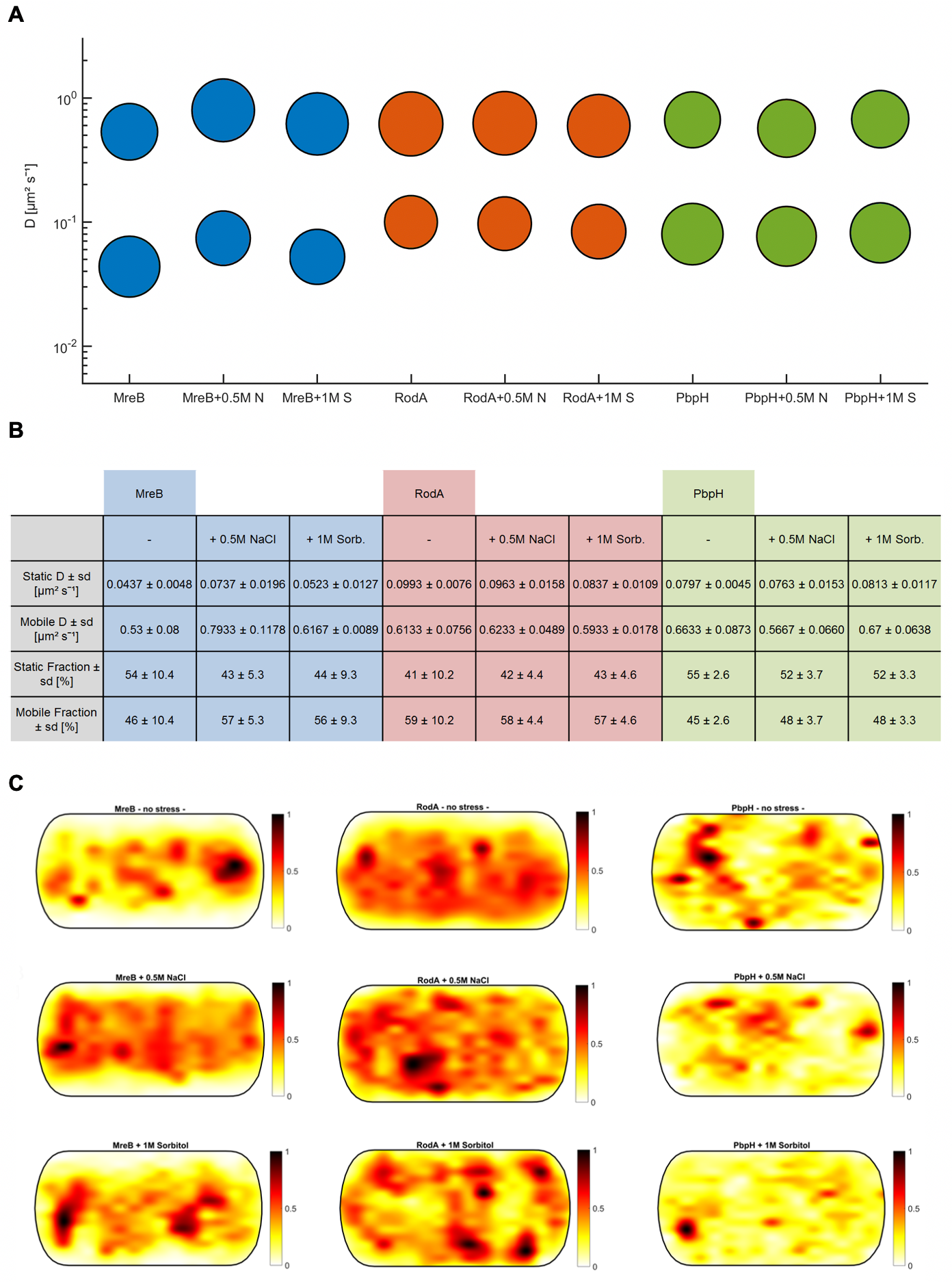

Supplement: FIGURE S5 — (A) Bubble plot of diffusion and fraction size for MreB, RodA, and PbpH under different stress conditions and (B) corresponding table. (C) Heat maps of the probabilistic distribution of tracks in a normalized cell for MreB, RodA, and PbpH after addition of 0.5 M NaCl or 1 M Sorbitol (dark red: higher probability, white: lower probability). [file Image_5.tiff]

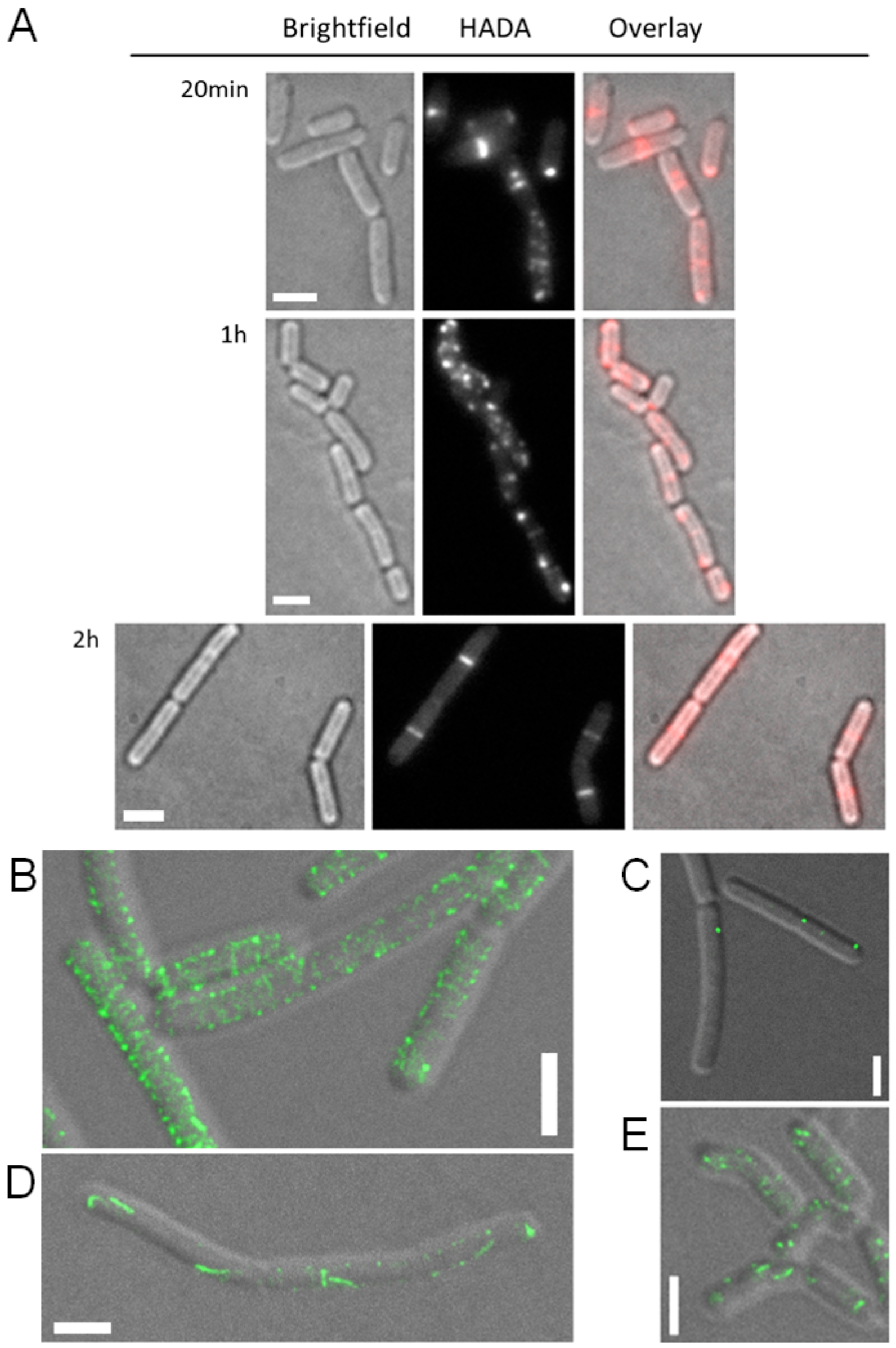

Supplement: FIGURE S6 — (A) Bright field, HADA (red-channel, 0.5 mM) and overlay images of B. subtilis PY79 cells in exponential phase in S750 media: 20 min HADA with added 0.5 M NaCl after 20 min, 1, 2 h; images taken after washing three times with PBS, scale bar 2 μm; (B–E) Gated-STED image of YFP-MreB under the control of the xylose promotor (+0.01% Xyl) in S750 media (green), overlaid on a DIC image of the cells after (B) exponentially growing cells, (C) 1 h of incubation without shaking, (D) 2 h of 0.5 M NaCl addition, (E) 5 min 50° heat shock, scale bars 2 μm. [file Image_6.TIF]

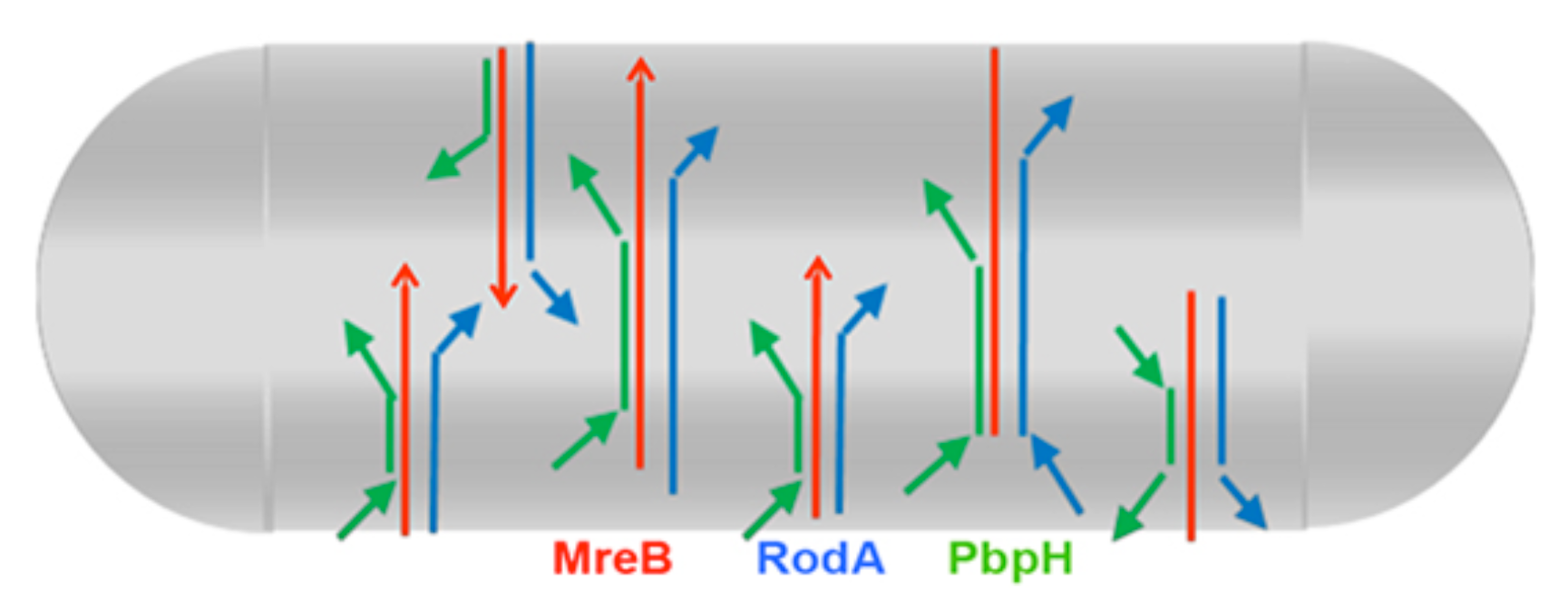

Supplement: FIGURE S7 — Illustration of the measured trajectory lengths in the bacteria, PbpH (green) exchanges with the PG synthesis machinery significantly faster than RodA (blue), while both move less persistently than MreB (red). [file Image_7.TIF]
